# Severe Maternal Morbidity Associated With Chronic Hypertension, Preeclampsia, and Gestational Hypertension

**DOI:** 10.1001/jamanetworkopen.2024.51406

**Published:** 2025-01-28

**Authors:** Erica P. Gunderson, Mara Greenberg, Michael Najem, Baiyang Sun, Stacey E. Alexeeff, Janet Alexander, Mai N. Nguyen-Huynh, James M. Roberts

**Affiliations:** 1 Division of Research, Kaiser Permanente Northern California, Pleasanton; 2 Department of Health Systems Science, Kaiser Permanente Bernard J. Tyson School of Medicine, Pasadena, California; 3 Department of Obstetrics and Gynecology, Kaiser Permanente Northern California, Oakland Medical Center, Oakland; 4 Department of Clinical Science, Kaiser Permanente Bernard J. Tyson School of Medicine, Pasadena, California; 5 Department of Neurology, Kaiser Permanente, Walnut Creek Medical Center, Walnut Creek, California; 6 Magee-Womens Research Institute, Department of Obstetrics, Gynecology and Reproductive Sciences, Epidemiology and Clinical and Translational Research, University of Pittsburgh, Pittsburgh, Pennsylvania

## Abstract

**Importance:**

Chronic hypertension and preeclampsia are leading risk enhancers for maternal-neonatal morbidity and mortality. Severe maternal morbidity (SMM) indicators include heart, kidney, and liver disease, but studies have not excluded patients with preexisting diseases that define SMM. Thus, SMM risks for uncomplicated chronic hypertension specific to preeclampsia remain unclear.

**Objective:**

To determine SMM rates and estimate relative risks associated with hypertensive disorders of pregnancy among patients with and without chronic hypertension unencumbered by preexisting vascular or end organ diseases.

**Design, Setting, and Participants:**

This retrospective cohort study used longitudinal health data from electronic health records from patients within a community-based, integrated health care system in northern California. The study cohort selected 263 518 pregnant patients without pregestational heart, kidney, or liver disease entering prenatal care at 14 weeks’ gestation or earlier and delivering a singleton stillbirth or live birth in 2009 to 2019. The data were analyzed between February 2022 and March 2024.

**Exposures:**

Five joint subgroups combining chronic hypertension status and the hypertensive disorders developing during pregnancy, defined as follows: (1) chronic hypertension with superimposed preeclampsia, (2) chronic hypertension and no preeclampsia, (3) no chronic hypertension with preeclampsia, (4) gestational hypertension, and (5) no chronic hypertension and no preeclampsia or gestational hypertension (reference group).

**Main Outcomes and Measures:**

The main outcome was SMM rates at delivery hospitalization (cases per 10 000 births) using the Centers for Disease Control and Prevention criteria (≥1 of 21 indicators to define SMM) obtained from electronic health records. Modified Poisson regression models estimated crude and adjusted relative risks (aRRs) and 95% CIs of SMM associated with the chronic hypertension and developing hypertensive disorders of pregnancy groups vs the reference group (no chronic hypertension and no preeclampsia or gestational hypertension) adjusted for clinical, sociodemographic, social, and behavioral covariates.

**Results:**

The analysis included a total of 263 518 pregnant patients (mean [SD] age at delivery, 31.0 [5.3] years), including 249 892 patients without chronic hypertension (4.7% developed preeclampsia) and 13 626 patients with chronic hypertension (31.5% developed superimposed preeclampsia). The highest SMM rates occurred in the no chronic hypertension with preeclampsia (934.3 [95% CI, 882.3-988.3] cases per 10 000 births) and the chronic hypertension with superimposed preeclampsia (898.3 [95% CI, 814.5-987.8] cases per 10,000 births) groups. Lower SMM rates occurred in the chronic hypertension and no preeclampsia (195.1 [95% CI, 168.0-225.2] cases per 10,000 births), gestational hypertension (312.7 [95% CI, 281.6-346.1] cases per 10,000 births), and no chronic hypertension and no preeclampsia or gestational hypertension (165.8 [95% CI, 160.6-171.2] cases per 10,000 births) groups *(P* < .001). Compared with the no chronic hypertension and no preeclampsia or gestational hypertension group, risks of SMM were significantly higher for the chronic hypertension with superimposed preeclampsia group (aRR, 4.97 [95% CI, 4.46-5.54]), no chronic hypertension with preeclampsia group (aRR, 5.12 [95% CI, 4.79-5.48]), chronic hypertension and no preeclampsia group (aRR, 1.17 [95% CI, 1.003-1.36]; *P* = .046), and the gestational hypertension group (aRR, 1.78 [95% CI 1.60-1.99]).

**Conclusions and Relevance:**

This cohort study found that the highest SMM rates at delivery hospitalization occurred for preeclampsia superimposed on chronic hypertension and preeclampsia without chronic hypertension, while gestational hypertension had intermediate rates of SMM. The patients with chronic hypertension who did not develop preeclampsia had SMM rates that were nearly the same as the lowest-risk patients without chronic hypertension who did not develop preeclampsia or gestational hypertension. These findings provide evidence that prevention of preeclampsia among patients with uncomplicated chronic hypertension is paramount to mitigating maternal morbidity.

## Key Points

**Question:**

Does severe maternal morbidity (SMM) among patients without preexisting end organ disease differ according to chronic hypertension status with and without preeclampsia compared to patients without chronic hypertension, or no hypertensive disorders developing during pregnancy?

**Findings:**

Among 263 518 patients without preexisting heart, kidney, or liver disease, preeclampsia developed in 31.5% with and 4.7% without chronic hypertension. The SMM risk was 5 times higher for chronic hypertension with superimposed preeclampsia and for preeclampsia alone, and almost 2-fold higher for gestational hypertension, while chronic hypertension without preeclampsia had nearly the same SMM risk as the lowest risk group of patients with no chronic hypertension and no hypertensive disorders developing during pregnancy.

**Meaning:**

In pregnant patients with uncomplicated chronic hypertension, prevention of preeclampsia may potentially reduce SMM risk comparable to normotensive patients.

## Teaser

This cohort study evaluates severe maternal morbidity risk for patients with and without chronic hypertension according to hypertensive disorders developing during pregnancy, unencumbered by preexisting vascular, or end organ diseases.

## Introduction

Hypertension complicating pregnancy is among the leading causes of maternal-fetal morbidity and mortality^1,2^ and increased risk of cardiovascular disease after pregnancy.^3^ In US women of reproductive age, chronic hypertension has been steadily increasing during pregnancy, in step with maternal obesity.^4,5^ The American College of Obstetricians and Gynecologists (ACOG), and the American Heart Association define chronic hypertension during pregnancy as systolic blood pressure (SBP) of 140 mm Hg or greater and/or diastolic BP (DBP) of 90 mm Hg or greater prior to pregnancy or diagnosed before 20 weeks’ gestation.^1,6^ This condition affects approximately 3% to 5% of pregnancies.^7^ Hypertensive disorders of pregnancy, including preeclampsia and gestational hypertension, affect 8% to 10% of patients without chronic hypertension, while superimposed preeclampsia affects 20% to 40% of patients with chronic hypertension.^8^ Hypertensive disorders of pregnancy combined with pregestational comorbidities substantially exacerbate maternal morbidity and mortality.^9^ Because chronic hypertension is relatively uncommon in female individuals of reproductive age, studies of severe maternal morbidity (SMM) have rarely evaluated risk for patients with chronic hypertension before or during pregnancy or did not exclude preexisting diseases and relied on limited administrative or vital statistics data.^10,11^ Thus, the risk of maternal morbidity outcomes for patients with chronic hypertension, complicated or not by preeclampsia and in the absence of other preexisting disease, remains unclear.

The Centers for Disease Control and Prevention (CDC) defines SMM as having 1 or more of 21 clinical indicators of serious complications related to pregnancy at delivery hospitalization.^12^ In the US, SMM occurs in 0.5% to 3.0% of births and has increased by 200% from 1993 to 2014, with 1.8% of births affected in 2021.^9,13^ Maternal risk enhancers of SMM include prepregnancy obesity, advanced maternal age, multifetal gestation, smoking habit, and government insurance.^14,15,16^ Pregestational heart, kidney, or liver disease complicate approximately 0.5% to 1% of all pregnancies and have low prevalence among young adults with chronic hypertension (approximately 2%-4%).^17^ One study found a 6-fold higher risk of acute renal failure with chronic hypertension vs no chronic hypertension but did not discern risk differences for preexisting disease or preeclampsia severity.^17^ Second, pregestational comorbidities have been conflated with the SMM indicators, such that preexisting disease would inflate SMM risk estimates. Finally, studies of SMM have generally adjusted for chronic hypertension (yes or no), and/or aggregated pregestational morbidities as potential confounders, including preexisting hypertension and other morbidities prior to pregnancy that are among the 21 indicators used to define SMM.^18,19^ Thus, the burden of SMM for chronic hypertension uncomplicated by prior heart, kidney, or liver disease according to developing hypertension during pregnancy remains unclear.

This study sought to determine the SMM rates at delivery hospitalization associated with chronic hypertension and hypertensive disorders developing during pregnancy without preexisting disease diagnoses from electronic health records (EHR) for pregnancies ending in live birth or stillbirth within a single, integrated, community-based health care system in northern California. We hypothesized that, when accounting for lifestyle behaviors and clinical, social, and demographic factors in patients without pregestational diseases defining SMM outcomes, (1) absolute rates and relative risks (RRs) of SMM would be higher for preeclampsia with chronic hypertension than for preeclampsia without chronic hypertension and (2) absolute rates and RRs of SMM would be intermediate for chronic hypertension without preeclampsia and for gestational hypertension compared to no chronic hypertension without hypertensive disorders of pregnancy. By evaluating the spectrum of hypertensive disorders that occur both before and during pregnancy for patient subgroups, we can estimate the true risks of SMM for women with chronic hypertension uncomplicated by preexisting end organ or vascular disease relative to preeclampsia.

## Methods

This cohort study was approved by the Kaiser Permanente Northern California (KPNC) institutional review board, which waived the requirement for informed consent from patients, given the minimal risk for this data only study design. This study is reported following the Strengthening the Reporting of Observational Studies in Epidemiology (STROBE) reporting guideline.

### Research Setting

KPNC is an integrated health care delivery system providing care to more than 4.6 million members through more than 10 000 physicians, more than 255 medical facilities, and 21 hospitals. The KPNC service area spans 22 counties of the greater Bay Area, as well as the California central valley from Fresno to Roseville and Sacramento. The 16 KPNC hospitals with labor and delivery units deliver approximately 48 000 births per year, with prenatal care standardized across the medical centers and offices and 89.5% of pregnant individuals entering prenatal care before 14 weeks’ gestation.

The KPNC EHR system provides comprehensive data on clinical outpatient BP measurements, medical diagnoses, prepregnancy body mass index (BMI; calculated as weight in kilograms divided by height in meters squared), pregnancy course and outcomes, sociodemographic characteristics, and social and lifestyle factors for stillbirth or live birth delivered from January 1, 2009, to December 31, 2019.

### Study Design and Sample

This retrospective cohort study design included pregnant patients without comorbidities prior to pregnancy (ie, heart, kidney, or liver disease or malignant neoplasm) who entered KPNC prenatal care by 14 weeks’ gestation and delivered a singleton stillbirth or live birth (one singleton birth per individual). Among 271 787 pregnant individuals, we excluded 3986 with preexisting morbidities, 47 missing all BP measurements, and 4322 with a history of preeclampsia. The final sample included 263 518 patients without previously specified morbidities, yielding 249 892 pregnant patients without chronic hypertension or history of preeclampsia and 13 626 pregnant patients with chronic hypertension (eFigure 1 in Supplement 1). Of these, 1.27% of patients with chronic hypertension and 1.02% of patients without chronic hypertension were excluded with preexisting heart, kidney, or liver diseases.

### SMM Outcome Definition

SMM was defined by the CDC criteria as the presence of at least 1 of 21 indicators (eTable 1 in Supplement 1) for the index birth at the delivery hospitalization using the specific *International Classification of Diseases, Ninth Revision *(*ICD-9*) and *International Statistical Classification of Diseases and Related Health Problems, Tenth Revision (ICD-10)* codes and procedure codes (eTable 2 in Supplement 1). Additionally, we included KPNC local-use procedure codes for cardiac rhythm, temporary tracheostomy, ventilation, and hysterectomy indicators. All codes were obtained from the EHR, including outpatient encounters and inpatient hospital discharge summaries to calculate overall rates of SMM as cases per 10 000 births.^12,20^

### Chronic Hypertension Before Pregnancy and/or During the First 20 Weeks of Gestation

Chronic hypertension was identified before pregnancy and during the first half of gestation as previously described.^20,21^ Prepregnancy chronic hypertension was defined as hypertension identified up to 2 years before conception of the index birth using a validated algorithm applied to the patient’s EHR^22^: presence of *ICD-9* or *ICD-10* codes for hypertension identified on 2 separate dates, BP measurements for stage 2 hypertension, BP elevations (SBP ≥140 mm Hg or DBP ≥90 mm Hg) from outpatient records on 2 separate consecutive days at least 3 months apart, or *ICD-9* or *ICD-10* codes for chronic hypertension plus a dispensed prescription for antihypertensive therapy. We also identified chronic hypertension during 0 to 20 weeks of gestation based on stage 2 BP elevations (on 2 separate days), with or without use of antihypertensive medications, or from *ICD-9* or *ICD-10* codes for chronic hypertension diagnosis before or during early pregnancy.

### Hypertensive Disorders Developing During Pregnancy: Preeclampsia and Gestational Hypertension

Hypertensive disorders of pregnancy include SBP elevations greater than or equal to 140 mm Hg or DBP elevations greater than or equal to 90 mm Hg with or without organ dysfunction that develops after 20 weeks’ gestation per the 2013 ACOG criteria.^23^ In this study, hypertensive conditions were identified throughout gestation from the EHR system and validated from EHR review as previously detailed.^20^ Briefly, we classified preeclampsia (BP elevation with proteinuria and/or organ dysfunction), and gestational hypertension (BP elevation without proteinuria or organ dysfunction) via *ICD-9* or *ICD-10* codes via our validated EHR algorithm including the gestational age of the diagnosis. The validation study showed high accuracy of the *ICD-9* and *ICD-10* codes to correctly classify hypertensive disorders, with sensitivity and specificity of 94% for preeclampsia and sensitivity and specificity of more than 85% and 91%, respectively, for gestational hypertension.^20^

### Preexisting Chronic Hypertension and Developing Hypertensive Disorders During Pregnancy

Pregnancy hypertensive conditions include both preexisting hypertension and hypertensive disorders that develop during pregnancy. They can occur as chronic hypertension with superimposed preeclampsia (BP elevation and new development of thrombocytopenia, liver dysfunction, renal insufficiency, or symptoms suggestive of preeclampsia), chronic hypertension without preeclampsia, preeclampsia in the absence of chronic hypertension, and gestational hypertension (BP elevation after 20 weeks’ gestation without end organ dysfunction).^24^

To evaluate the independent associations for chronic hypertension status and developing pregnancy disorders, we evaluated 2 separate variables: (1) chronic hypertension status, defined as chronic hypertension vs no chronic hypertension (reference group), and (2) hypertensive disorders developing during pregnancy, defined as any preeclampsia (with or without chronic hypertension), gestational hypertension (yes or no) vs no preeclampsia or gestational hypertension (with or without chronic hypertension) (reference group). Next, to evaluate the joint associations for preexisting and developing hypertensive disorders, we categorized 5 mutually exclusive subgroups for combinations of preexisting and developing hypertensive disorders during pregnancy as follows: (1) chronic hypertension with superimposed preeclampsia, (2) chronic hypertension and no preeclampsia, (3) no chronic hypertension with preeclampsia, (4) gestational hypertension, and (5) no chronic hypertension and no preeclampsia or gestational hypertension (reference group).

### Model Covariates

We obtained standard clinical and sociodemographic risk factors from the EHR: maternal age, parity, self-reported race and ethnicity (including American Indian or Alaskan Native, Asian, Black, Hispanic, multiple or unspecified, Native Hawaiian or Pacific Islander, and White), measured prepregnancy weight within 1 year prior to conception or weight at less than 14 weeks’ gestation and height to calculate BMI, gestational age at delivery, mode of delivery, and diabetes (gestational diabetes, pregestational diabetes vs neither). We also obtained lifestyle behaviors, including tobacco smoking habit during pregnancy (never, current, or former). Social factors included employer, self-funded, other type of insurance, or government health insurance (ie, Medicaid, MediCal, or state-subsidized), and the neighborhood deprivation index (NDI), based on the US Census Bureau’s American Community Survey data.^25^

Racial and ethnic identities represent multifactorial characteristics, such as shared history, language, beliefs, and customs, that may be influenced by social determinants of health (eg, education, socioeconomic disadvantage, structural racism, and discrimination).^26^ Jointly, these factors may contribute to disparities in health outcomes and have broad importance in health research for not only identifying but monitoring, understanding, and intervening on risk factors to ameliorate health inequities.^27^

### Statistical Analysis

We first computed the crude SMM rates per 10 000 births and 95% CIs overall and stratified by chronic hypertension status for the types of hypertensive disorders developing during pregnancy. We calculated the risk differences in SMM rates (with 95% CIs) according to the specific reference groups. We then fit modified Poisson regression models with robust standard errors to calculate the unadjusted RRs and adjusted RRs (aRRs) and 95% CIs of SMM for the associations with 2 separate variables: (1) chronic hypertension status (dichotomized), and (2) the hypertensive disorders developing during pregnancy.^28^ Model 1 estimated the crude (unadjusted) RR for chronic hypertension vs no chronic hypertension (reference group). Model 2 estimated the crude RRs for pregnancy hypertensive disorders categories: (1) any preeclampsia (with or without chronic hypertension), (2) gestational hypertension, and (3) no preeclampsia or gestational hypertension (with or without chronic hypertension) (reference group). Next, these 2 separate categorical variables were included in a single model to estimate their independent associations with the aRRs of SMM (model 3). Finally, model 4 estimated the fully adjusted aRR of SMM for all covariates added to model 3. Covariate selection was based on a priori criteria and included parity, maternal age, prepregnancy BMI, diabetes status, smoking habit, type of health insurance, racial and ethnic groups, and NDI. We then evaluated the interaction for chronic hypertension and preeclampsia within the SMM association model. We further evaluated additive interaction by computing the relative excess risk due to interaction. We also constructed a directed acyclic graph (DAG) to reflect the potential mediating relationship of preeclampsia on the pathway of chronic hypertension to SMM (eFigure 2 in Supplement 1). We conducted causal mediation analyses with and without exposure-mediator interaction following the methods of VanderWeele.^29^

In regression models for the 5 joint chronic hypertension and hypertensive disorders developing during pregnancy subgroups, models 1C and 2C (C signifies models for the 5 combined subgroups) estimated the unadjusted RR and aRR of SMM compared with no chronic hypertension and no preeclampsia or gestational hypertension (reference group). A sensitivity analysis stratified the sample by parity groups, with estimates of aRR of SMM for primiparas and multiparas in separate models. In secondary analyses, we repeated models 1C and 2C to estimate the aRR of the SMM outcome without the blood transfusion indicator. All *P* values were 2-sided with statistical significance set at *P* < .05. For interaction *P* values, statistical significance was set at *P* < .10. Analyses were conducted in SAS software version 9.4 (SAS Institute). The data were analyzed between February 2022 and March 2024.

## Results

The study cohort consisted of 263 518 pregnant patients with or without a diagnosis of chronic hypertension and free of prior heart, liver, or kidney disease or malignant neoplasm who delivered a singleton live birth or stillbirth from 2009 to 2019 (Table 1). Of these, 13 626 were diagnosed with chronic hypertension (5.2%), of whom 4297 (31.5%) developed superimposed preeclampsia. Among patients in the no chronic hypertension group, 11 744 (4.7%) developed preeclampsia, 11 514 (4.6%) developed gestational hypertension, and 226 604 (90.7%) did not develop preeclampsia or gestational hypertension. The distribution of racial and ethnic groups was 0.3% American Indian or Alaskan Native, 25.1% Asian, 7.6% Black, 26.3% Hispanic, 3.4% multiple or unspecified race and ethnicity, 0.7% Native Hawaiian or Pacific Islander, and 36.6% White.

**Table 1.** Maternal Characteristics by SMM Overall and Stratified by Chronic Hypertension Status

Characteristic	Patients, No. (%)					
	Overall (N = 263 518)		Chronic hypertension (n = 13 626)		No chronic hypertension (n = 249 892)	
	SMM	No SMM	SMM	No SMM	SMM	No SMM
Overall	5786 (2.2)	257 732 (97.8)	568 (4.2)	13 058 (95.8)	5218 (2.1)	244 674 (97.9)
Age, y						
18-25	1034 (17.9)	46 087 (17.9)	64 (11.3)	1305 (10.0)	970 (18.6)	44 782 (18.3)
26-30	1570 (27.1)	79 653 (30.9)	109 (19.2)	3278 (25.1)	1461 (28.0)	76 375 (31.2)
31-35	1874 (32.4)	86 207 (33.4)	185 (32.6)	4555 (34.9)	1689 (32.4)	81 652 (33.4)
36-40	1055 (18.2)	38 662 (15.0)	163 (28.7)	3088 (23.6)	892 (17.1)	35 574 (14.5)
41-45	253 (4.4)	7123 (2.8)	47 (8.3)	832 (6.4)	206 (3.9)	6291 (2.6)
Race and ethnicity						
American Indian or Native Alaskan	14 (0.2)	809 (0.3)	0	43 (0.3)	14 (0.3)	766 (0.3)
Asian	1705 (29.5)	64 385 (25.0)	133 (23.4)	2547 (19.5)	1572 (30.1)	61 838 (25.3)
Black	510 (8.8)	19 475 (7.6)	90 (15.8)	1829 (14.0)	420 (8.0)	17 646 (7.2)
Hispanic	1516 (26.2)	67 781 (26.3)	151 (26.6)	3143 (24.1)	1365 (26.2)	64 638 (26.4)
Multiple or not specified	180 (3.1)	8703 (3.4)	14 (2.5)	410 (3.1)	166 (3.2)	8293 (3.4)
Native Hawaiian or Pacific Islander	56 (1.0)	1916 (0.7)	12 (2.1)	115 (0.9)	44 (0.8)	1801 (0.7)
White	1805 (31.2)	94 663 (36.7)	168 (29.6)	4971 (38.1)	1637 (31.4)	89 692 (36.7)
Prenatal parity						
Nulliparous (0 births)	3993 (69.0)	145 956 (56.6)	325 (57.2)	6752 (51.7)	3668 (70.3)	139 204 (56.9)
Primiparous (1 birth)	1085 (18.8)	69 691 (27.0)	124 (21.8)	3458 (26.5)	961 (18.4)	66 233 (27.1)
Biparous (2 births)	434 (7.5)	28 575 (11.1)	67 (11.8)	1717 (13.1)	367 (7.0)	26 858 (11.0)
Multiparous (≥3 births)	274 (4.7)	13 510 (5.2)	52 (9.2)	1131 (8.7)	222 (4.3)	12 379 (5.1)
Diabetes status						
Pregestational diabetes	113 (2.0)	2357 (0.9)	57 (10.0)	855 (6.5)	56 (1.1)	1502 (0.6)
Gestational diabetes	821 (14.2)	30 886 (12.0)	136 (23.9)	3054 (23.4)	685 (13.1)	27 832 (11.4)
None	4852 (83.9)	224 489 (87.1)	375 (66.0)	9149 (70.1)	4477 (85.8)	215 340 (88.0)
Prepregnancy BMI						
<18.5	173 (3.0)	6647 (2.6)	3 (0.5)	56 (0.4)	170 (3.3)	6591 (2.7)
18.5-24.9	2635 (45.5)	123 275 (47.8)	106 (18.7)	2606 (20.0)	2529 (48.5)	120 669 (49.3)
25-29.9	1527 (26.4)	69 121 (26.8)	136 (23.9)	3281 (25.1)	1391 (26.7)	65 840 (26.9)
30-34.9	819 (14.2)	32 779 (12.7)	152 (26.8)	2882 (22.1)	667 (12.8)	29 897 (12.2)
35-39.9	349 (6.0)	14 645 (5.7)	82 (14.4)	2030 (15.5)	267 (5.1)	12 615 (5.2)
≥40	250 (4.3)	9418 (3.7)	86 (15.1)	2147 (16.4)	164 (3.1)	7271 (3.0)
Unknown	33 (0.6)	1847 (0.7)	3 (0.5)	56 (0.4)	30 (0.6)	1791 (0.7)
NDI						
≤−1 (least deprived)	647 (11.2)	29 274 (11.4)	56 (9.9)	1142 (8.7)	591 (11.3)	28 132 (11.5)
>−1 to 0	2882 (49.8)	129 785 (50.4)	256 (45.1)	6464 (49.5)	2626 (50.3)	123 321 (50.4)
>0 to 1	1498 (25.9)	67 050 (26.0)	163 (28.7)	3643 (27.9)	1335 (25.6)	63 407 (25.9)
>1 (most deprived)	747 (12.9)	31 206 (12.1)	91 (16.0)	1786 (13.7)	656 (12.6)	29 420 (12.0)
Unknown	12 (0.2)	417 (0.2)	2 (0.4)	23 (0.2)	10 (0.2)	394 (0.2)
Type of health insurance						
Government: MediCal, Medicaid, or state-subsidized	495 (8.6)	19 129 (7.4)	57 (10.0)	1166 (8.9)	438 (8.4)	17 963 (7.3)
Employer, self-funded, or other	5291 (91.4)	238 603 (92.6)	511 (90.0)	11 892 (91.1)	4780 (91.6)	226 711 (92.7)
Smoking status						
Current	316 (5.5)	14 823 (5.8)	41 (7.2)	1011 (7.7)	275 (5.3)	13 812 (5.6)
Former	723 (12.5)	31 813 (12.3)	84 (14.8)	2043 (15.6)	639 (12.2)	29 770 (12.2)
Never	4719 (81.6)	209 827 (81.4)	442 (77.8)	9935 (76.1)	4277 (82.0)	199 892 (81.7)
Unknown	28 (0.5)	1269 (0.5)	1 (0.2)	69 (0.5)	27 (0.5)	1200 (0.5)

Abbreviations: BMI, body mass index (calculated as weight in kilograms divided by height in meters squared); NDI, Neighborhood Deprivation Index; SMM, severe maternal morbidity.

### SMM Rates for Hypertensive Disorders of Pregnancy 

**Figure 1.**
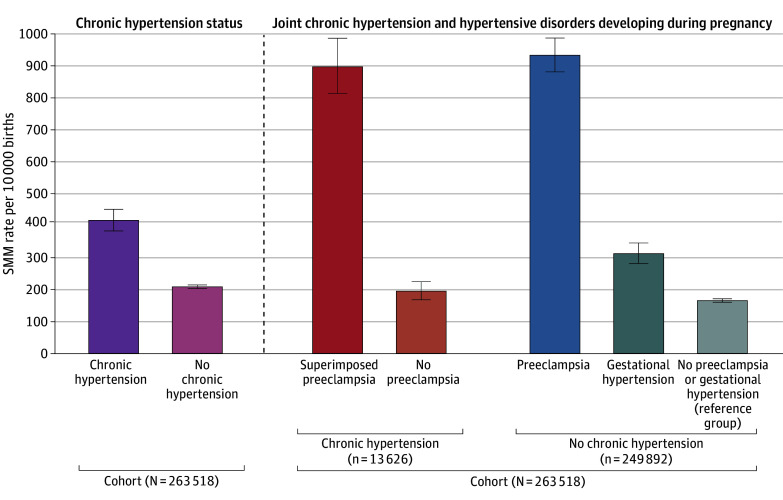
Overall Severe Maternal Morbidity Rates Per 10 000 Births by Chronic Hypertension Status, and 5 Joint Chronic Hypertension and Hypertensive Disorders Developing During Pregnancy Subgroups Gestational hypertension does not apply to the chronic hypertension group. Error bars indicate 95% CIs.

There were 5786 SMM cases, for an overall rate of 219.6 (95% CI, 214.0-225.2) per 10 000 births. The SMM rates varied by diagnoses of hypertensive disorders both before and during pregnancy (Figure 1). The SMM rate per 10 000 births was 2 times higher for patients with chronic hypertension (416.9 [95% CI, 383.9-451.8]) than for those without chronic hypertension (208.8 [95% CI, 203.2-214.5]) (*P* < .001) without considering preeclampsia conditions or gestational hypertension. The SMM rates per 10 000 births were highest for groups with and without chronic hypertension who developed preeclampsia, at 898.3 (95% CI, 814.5-987.8) for the chronic hypertension with superimposed preeclampsia group and 934.3 (95% CI, 882.3-988.3) for the no chronic hypertension with preeclampsia group. SMM rates were lowest for patients without chronic hypertension who did not develop preeclampsia or gestational hypertension. In the chronic hypertension and no preeclampsia group, the SMM rate per 10 000 births was 195.1 (95% CI, 168.0-225.2), which was similar in magnitude to the no chronic hypertension and no preeclampsia or gestational hypertension (reference) group (165.8 [95% CI, 160.6-171.2]) (*P* < .001) and significantly lower than for the rate for the gestational hypertension group (312.7 [95% CI, 281.6-346.1]) (all *P* < .001).

### Characteristics for Chronic Hypertension and SMM

Patients with chronic hypertension who developed SMM tended to be older, be nulliparous, have pregestational diabetes, have government health insurance, and self-report Asian, Black, Hispanic, or Native Hawaiian or Pacific Islander race and ethnicity compared to those who did not develop SMM (Table 1). Among patients who developed SMM, those with chronic hypertension were older, less likely to report Asian race, and more likely to be current smokers, primiparous or multiparous, to have obesity, diabetes, or government health insurance, and had greater economic deprivation than those without chronic hypertension.

### Characteristics for Preexisting Chronic Hypertension Groups

Patients with chronic hypertension, regardless of superimposed preeclampsia (eTable 3 in Supplement 1), were older and more likely to be primiparous or multiparous; were more likely to have obesity, pregestational diabetes, gestational diabetes, earlier delivery, greater deprivation, and government health insurance; and were more likely to be former or current smokers and to report Black race compared with no chronic hypertension groups. Patients with gestational hypertension or preeclampsia were more likely to be younger; to be nulliparous; to report Hispanic ethnicity; to report Black or White race; and to have obesity, pregestational diabetes, gestational diabetes, and higher gestational weight gain compared with patients with no chronic hypertension and no hypertensive disorders developing during pregnancy; they were also more likely to be current or former smokers. Patients who developed preeclampsia were more likely to have cesarean delivery and to deliver before 37 weeks’ gestation (eTable 4 in Supplement 1).

### Models of the Relative Risk of SMM for the Two Independent Variables 

In separate regression models for each of the 2 independent variables for hypertensive disorders of pregnancy (Table 2; eTable 5 in Supplement 1), the unadjusted RRs of SMM were 2.00 (95% CI, 1.83-2.17) for the chronic hypertension vs no chronic hypertension groups (model 1), 5.54 (95% CI, 5.23-5.86) for the any preeclampsia (with or without chronic hypertension) group, and 1.87 (95% CI, 1.68-2.08) for the gestational hypertension group compared with the no preeclampsia or gestational hypertension (with or without chronic hypertension) group (model 2). In model 3, the aRRs of SMM were 1.03 (95% CI, 0.94-1.13) for chronic hypertension vs no chronic hypertension groups, 5.49 (95% CI, 5.17-5.84) for the any preeclampsia (with or without chronic hypertension) group, and 1.87 (95% CI, 1.69-2.09) for gestational hypertension group vs no preeclampsia or gestational hypertension (with or without chronic hypertension) group (reference). In the fully adjusted model 4, the aRRs of SMM were similar to model 3: 1.04 (95% CI, 0.94-1.14) for chronic hypertension vs no chronic hypertension groups, 5.00 (95% CI, 4.69-5.34) for any preeclampsia (with or without chronic hypertension) group, and 1.77 (95% CI, 1.59-1.98) for the gestational hypertension vs no preeclampsia or gestational hypertension (with or without chronic hypertension) groups (reference). Causal mediation analyses (eTable 6 in Supplement 1) found statistically significant mediation of chronic hypertension by preeclampsia in models with (*P* < .001) and without (*P* = .04) exposure-mediator interaction.

**Table 2.** RRs of SMM for 2 Independent Variables and 5 Joint Chronic Hypertension and Hypertensive Disorders Developing During Pregnancy Subgroups

Hypertensive condition group	SMM cases/total group	Unadjusted and Adjusted RR (95% CI)			
Models: 2 independent variables for hypertensive disorders		Model 1 unadjusted	Model 2 unadjusted	Model 3 independently adjusted	Model 4 fully adjusted plus covariates^a^
	No./total No. (%)	Unadjusted RR (95% CI)	Unadjusted RR (95% CI)	aRR (95% CI)	aRR (95% CI)
1. Chronic hypertension status					
Chronic hypertension	568/13 626 (4.2)	2.00 (1.83-2.17)	NA	1.03 (0.94-1.13)	1.04 (0.94-1.14)
No chronic hypertension	5218/249 892 (2.1)	1.0 [Reference]	NA	1.0 [Reference]	1.0 [Reference]
2. Hypertensive disorders developing during pregnancy					
Any preeclampsia (with or without chronic hypertension)	1486/16 071 (9.2)	NA	5.54 (5.23-5.86)	5.49 (5.17-5.84)	5.00 (4.69-5.34)
Gestational hypertension	360/11 514 (3.1)	NA	1.87 (1.68-2.08)	1.87 (1.69-2.09)	1.77 (1.59-1.98)
No preeclampsia or gestational hypertension (with or without chronic hypertension)	3940/235 933 (1.7)	NA	1.0 [Reference]	1.0 [Reference]	1.0 [Reference]
**Models: 5 joint subgroups: ** **chronic hypertension and hypertensive disorders developing during pregnancy **	**SMM cases/total group **	**Model 1C** **unadjusted** ^b^	**NA**	**NA**	**Model 2C fully adjusted plus covariates** ^a^ ^,^ ^b^
	No./total No. (%)	Unadjusted RR (95% CI)			aRR (95% CI)
1. Chronic hypertension with superimposed preeclampsia	386/4297 (9.0)	5.42 (4.90-5.99)	NA	NA	4.97 (4.46-5.54)
2. Chronic hypertension and no preeclampsia	182/9329 (2.0)	1.18 (1.02-1.36)	NA	NA	1.17 (1.00-1.36)^c^
3. No chronic hypertension with preeclampsia	1100/11 774 (9.3)	5.63 (5.28-6.01)	NA	NA	5.12 (4.79-5.48)
4. Gestational hypertension	360/11 514 (3.1)	1.89 (1.69-2.10)	NA	NA	1.78 (1.60-1.99)
5. No chronic hypertension and no preeclampsia or gestational hypertension	3758/226 604 (1.7)	1.0 [Reference]	NA	NA	1.0 [Reference]

Abbreviations: aRR, adjusted rate ratio; NA, not applicable; RR, rate ratio; SMM, severe maternal morbidity.

^a^ Covariates: Racial and ethnic groups, age, parity, prepregnancy BMI, diabetes status, smoking habit, type of health insurance, and neighborhood deprivation index (NDI).

^b^ Interaction *P* values for 5 joint (C = combined) chronic hypertension and hypertensive disorders developing during pregnancy group (preeclampsia) associations: .032 without adjusting for covariates (Model 1C), and .052 after fully adjusting for covariates (Model 2C).

^c^ *P* = .046.

### Models of Relative Risk of SMM for the 5 Joint Subgroups 

By contrast, multivariable models for the 5 joint preexisting chronic hypertension, and hypertensive disorders developing during pregnancy subgroups (Table 2; eTable 7 in Supplement 1) estimated the unadjusted RRs (model 1C) and fully adjusted aRRs (model 2C) of SMM for each mutually exclusive subgroup. Compared with the no chronic hypertension and no preeclampsia or gestational hypertension group (reference), aRRs of SMM were 4.97 (95% CI, 4.46-5.54) for chronic hypertension with superimposed preeclampsia, and 5.12 (95% CI, 4.79-5.48) for no chronic hypertension with preeclampsia groups. The aRR of SMM for the chronic hypertension and no preeclampsia group was 1.17 (95% CI, 1.00-1.36; *P* = .046), while for the gestational hypertension group, it was 1.78 (95% CI, 1.60-1.99) compared with no chronic hypertension and no preeclampsia or gestational hypertension (reference group). Results were similar when these models were stratified by parity groups (eTable 8 in Supplement 1). There was evidence for a significant multiplicative interaction between chronic hypertension status and preeclampsia (interaction *P* = .05); (Table 2). There was no evidence of additive interaction between chronic hypertension status and preeclampsia (relative excess risk due to interaction, −0.36 [95% CI, −1.05 to 0.34]).

### Most Common Individual SMM Indicators 

**Figure 2.**
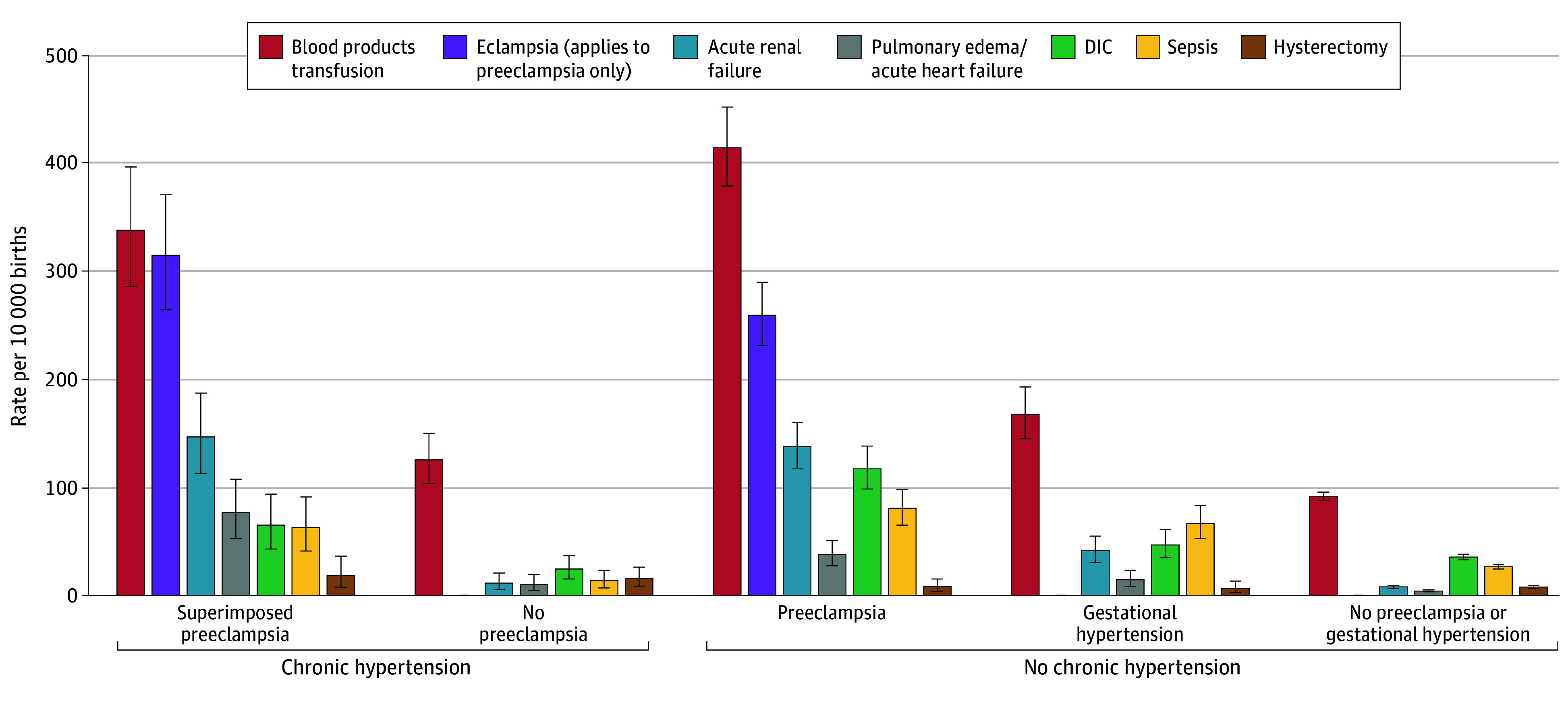
Top 7 Most Common Severe Maternal Morbidity Indicators Among 5 Joint Chronic Hypertension and Hypertensive Disorders Developing During Pregnancy Subgroups DIC indicates disseminated intravascular coagulation; error bars, 95% CIs.

The 7 most common individual SMM indicators were blood products transfusion, eclampsia, acute kidney failure, disseminated intravascular coagulation (DIC), pulmonary edema or acute heart failure, sepsis, and hysterectomy (eTable 1 in Supplement 1) based on the individual rates for each indicator (number of patients with the indicator per 10 000 births). The rates for each of the 7 SMM indicators varied among the 5 joint chronic hypertension and hypertensive disorders developing during pregnancy subgroups (Figure 2). For the chronic hypertension with superimposed preeclampsia group, blood products transfusion was highest, followed, in order, by eclampsia, acute kidney failure, pulmonary edema or acute heart failure, DIC, sepsis, and hysterectomy. The no chronic hypertension with preeclampsia group had a very similar pattern for SMM indicators, except for higher DIC and sepsis, and lower pulmonary edema or acute heart failure rates than for the chronic hypertension with superimposed preeclampsia group. These SMM indicator rates were substantially lower for the chronic hypertension and no preeclampsia group and the no chronic hypertension and no preeclampsia or gestational hypertension group and intermediate for the gestational hypertension group.

### SMM Rates Overall and Minus the Blood Transfusion Indicator 

Table 3 displays the SMM rates overall and rates minus the blood transfusion indicator for the 5 joint chronic hypertension and hypertensive disorders developing during pregnancy subgroups. The SMM cases without the blood transfusion indicator comprised 73.1% of the SMM cases for the chronic hypertension with superimposed preeclampsia group and 64.2% of cases for the no chronic hypertension with preeclampsia group. By contrast, the 20 indicators minus the blood transfusion indicator comprised lower percentages of SMM cases in the chronic hypertension and no preeclampsia (45.1%), gestational hypertension (54.2%), and no chronic hypertension and no preeclampsia or gestational hypertension (53.0%) groups. The risk differences showed greater absolute risk for each of the mutually exclusive subgroups of hypertensive disorders vs no chronic hypertension and no preeclampsia or gestational hypertension (eFigure 3 in Supplement 1). Finally, in multivariable models for SMM without blood transfusion, aRRs of SMM for chronic hypertension status and hypertensive disorders developing during pregnancy showed consistently stronger associations among patients with and without chronic hypertension who developed preeclampsia compared to the no chronic hypertension and no preeclampsia or gestational hypertension group (eTables 9-12 in Supplement 1).

**Table 3.** SMM Rates Overall and Without Blood Transfusions Among Chronic Hypertension and Joint Chronic Hypertension and Hypertensive Disorders Developing During Pregnancy Subgroups

Subgroup	Total sample size, No.	SMM rate		SMM rate without blood transfusion events		SMM cases minus transfusion, %
		No.	Cases per 10 000 births, No. (95% CI)	No.	Cases per 10 000 births, No. (95% CI)	
Chronic hypertension						
All	13 626	568	416.9 (383.9-451.8)	364	267.1 (240.7-295.6)	64.1
Superimposed preeclampsia	4297	386	898.3 (814.5-987.8)	282	656.3 (584.0-734.5)	73.1
No preeclampsia	9329	182	195.1 (168.0-225.2)	82	87.9 (70.0-109.0)	45.1
No chronic hypertension						
All	249 892	5218	208.8 (203.2-214.5)	2892	115.7 (111.6-120.0)	55.4
Preeclampsia	11 774	1100	934.3 (882.3-988.3)	706	599.6 (557.4-644.0)	64.2
Gestational hypertension	11 514	360	312.7 (281.6-346.1)	195	169.4 (146.6-194.6)	54.2
No preeclampsia or gestational hypertension	226 604	3758	165.8 (160.6-171.2)	1991	87.9 (84.1-91.8)	53.0
Total cohort^a^	263 518	5786	219.6 (214.0-225.2)	3256	123.6 (119.4-127.8)	56.3

Abbreviation: SMM, severe maternal morbidity.

^a^ Includes all individuals without heart, kidney, or liver disease or malignant neoplasms before or during pregnancy.

## Discussion

This cohort study evaluated a community-based sample of 263 518 patients without preexisting vascular or organ disease or malignant neoplasms and with early entry into prenatal care and delivery between 2009 and 2019 within a single integrated health care system in northern California. This study found that patients with and without chronic hypertension who developed preeclampsia both had comparable and much higher morbidity rates (per 10 000 births), and 5-fold higher relative risks of SMM compared to patients without chronic hypertension who did not develop preeclampsia or gestational hypertension, adjusted for clinical, social, and behavioral risk factors. The novel findings are that pregnant patients with chronic hypertension and no vascular or end organ disorders who avoided superimposed preeclampsia had only a 17% higher relative risk of SMM, while patients with gestational hypertension had an even greater 78% higher relative risk of SMM compared with patients with no hypertensive disorders. Furthermore, one-third of patients with chronic hypertension who developed preeclampsia experienced higher rates of the most serious SMM indicators, including cardiovascular, cerebrovascular, or renal failure events. For preeclampsia without chronic hypertension, heightened complications were marked by disorders related to blood transfusion (eg, DIC), and/or morbidities related to sepsis.

Previous studies have generally not addressed SMM outcomes specifically for patients with chronic hypertension because of smaller sample sizes and the lack of high-quality data systems to accurately capture preexisting disease conditions from administrative claims or vital statistics data systems. Studies using administrative databases or inpatient discharge records with linkage of clinical records have usually adjusted model estimates for chronic hypertension as a covariate to estimate the relative risk of SMM for chronic hypertension separately from any type of preeclampsia or gestational hypertension variable within the same model.^9,30,31^ Our models evaluating 2 separate variables found crude relative risks that were 5-fold higher for any preeclampsia (with or without chronic hypertension) and 2-fold higher for chronic hypertension that became null when the model included preeclampsia, which mediated more than 80% of the association of chronic hypertension with SMM.

To our knowledge, previous studies have not evaluated the SMM risk for chronic hypertension with superimposed preeclampsia separately from no chronic hypertension with preeclampsia. A previous tertiary center study of 7 025 pregnant individuals found a 2.1-fold higher crude odds of SMM in the 19 patients with SMM among 312 with chronic hypertension who did not develop preeclampsia. Yet, this study did not estimate risks for superimposed preeclampsia separately from preeclampsia alone, nor delineate preexisting disease or control for any potential confounders.^16^ Another study contrasted specific morbidity in those with chronic hypertension vs without chronic hypertension but did not evaluate overall risk of SMM or exclude patients with preexisting heart, kidney, or liver diseases from the analytic sample.^17^ A study^32^ of antihypertensive medication use in patients with prepregnancy hypertension found that 6.3% developed SMM and that risk varied by adherence to medication use. However, that study^32^ did not evaluate preeclampsia or underlying diseases prior to pregnancy. Prior studies did not distinguish clinical risk factors for SMM related to comorbidities for chronic hypertension. Pregestational diseases (ie, heart, kidney, or liver disorders) that are among the 21 indicators that define the SMM outcome cannot result from pregnancy and as such are not potential confounders.

### Strengths and Limitations

This study has several strengths, including the large community-based population receiving care within a single integrated health care system under standardized care protocols, comprehensive EHR data (including clinical and social factors), and racial and ethnic diversity. Validated algorithms identified 5% of the sample with chronic hypertension before and during pregnancy based on clinical data. The method used *ICD-9* and *ICD-10* codes plus longitudinal blood pressure measurements with or without antihypertensive medication use. Both preeclampsia and gestational hypertension diagnoses were validated with high sensitivity and specificity.^20^ Finally, the exacting methods also excluded patients with preexisting diseases that defined the outcome. These features reduced misclassification and involved standardized treatment protocols to reduce confounding. This sample was also representative of the US prenatal population, with 60% of patients from Asian, Black, and Hispanic groups.

This study also has some limitations. We did not evaluate BP levels or control under treatment during pregnancy in patients with or without chronic hypertension that may affect the risk of SMM. Economic deprivation was derived from neighborhood-level data instead of individual-level resources. Therefore, residual or unmeasured confounding may differentially affect the risks for patients with and without chronic hypertension. Furthermore, while KPNC members have less representation in the low and high economic strata, membership is representative of the regional diversity.

## Conclusions

This cohort study’s main finding is that preeclampsia accounted for nearly all of the excess risk of SMM associated with chronic hypertension in patients without concurrent preexisting heart, kidney, or liver disease predating pregnancy. For 70% of the patients with chronic hypertension who avoided superimposed preeclampsia, their relative risk of SMM was nearly the same, (only 17% higher) as that found in patients without chronic hypertension and no hypertensive disorders developing during pregnancy. We also found that patients who developed preeclampsia were 5 times more likely to experience SMM, which was the same relative risk for pregnant patients with and without chronic hypertension. Previous studies have not estimated SMM rates and relative risks related to preeclampsia stratified by chronic hypertension compared with normotensive women.

Several important questions remain unanswered. Although current management appears not to result in greater overall morbidity for chronic hypertension compared with no chronic hypertension according to preeclampsia outcome groups, there is little information on how BP, prenatal, and delivery strategies might prevent the development of preeclampsia. In fact, these guidelines have changed over the study period and continue to be areas of active investigation. The efficacy of early delivery or intense observations in patients with chronic hypertension can only be resolved by randomized clinical trials. The most important target for future studies is the prevention of preeclampsia.

The increasing trend for young adults to develop chronic hypertension before and during pregnancy is recognized as an urgent matter to forestall impending increased perinatal morbidity.^33,34^ We found that excess risk of SMM among individuals entering pregnancy with chronic hypertension without preexisting organ dysfunction was predominantly among the 30% of individuals who developed superimposed preeclampsia. Thus, current management is most appropriately directed at preventing preeclampsia in these patients at higher risk. Although there is some controversy about the efficacy of low-dose aspirin therapy to reduce the risk of preeclampsia in individuals with chronic hypertension,^35^ treatment is safe and should be administered as recommended by the ACOG and the Society of Maternal Fetal Medicine.^36^ Furthermore, healthful lifestyle behaviors, including sleep, diet, and physical activity, confer health benefits and should be supported in concert with follow-up care for those at high risk.^33^ Finally, early risk stratification for preeclampsia based on preexisting organ dysfunction, especially with chronic hypertension, is needed to identify subgroups for greater responsiveness to interventions.

This study fills an important evidence gap in showing that the absolute SMM risk for individuals with chronic hypertension who avoided preeclampsia was only modestly higher compared with individuals who remained normotensive. It also adds to the evidence to advance precision medicine to improve risk stratification for preeclampsia that is called for by professional organizations.^37,38,39^ Lastly, preeclampsia is the major driver of maternal and newborn morbidity and mortality. These findings underscore the urgency of prevention and control of chronic hypertension before and during pregnancy as an important strategy to mitigate morbidity due to preeclampsia in pregnancy.

## Notes

Supplement 1. **eTable 1. **Severe Maternal Morbidity Rates at Delivery Hospitalization Overall and for the 21 Individual Indicators by Chronic Hypertension Status and Stratified by Hypertensive Disorders Developing During Pregnancy **eTable 2.** Definition of Severe Maternal Morbidity Indicators. **eTable 3.** Maternal Characteristics by Chronic Hypertension Status and Hypertensive Disorders Developing During Pregnancy **eTable 4.** Gestational Age at Delivery, Prevalence of Preterm Deliveries, and Mode of Delivery According to Chronic Hypertension and Hypertensive Disorders Developing During Pregnancy **eTable 5.** Model 4: Fully Adjusted Relative Risks of Severe Maternal Morbidity for 2 Independent Variables for Hypertensive Disorders: Chronic Hypertension and Hypertensive Disorders Developing During Pregnancy With Covariates **eTable 6.** Causal Mediation Analysis **eTable 7.** Model 2C: Fully Adjusted Relative Risks of Severe Maternal Morbidity for 5 Joint Chronic Hypertension and Hypertensive Disorders Developing During Pregnancy Subgroups With Covariates **eTable 8.** Models 2C.1 and 2C.2: Fully Adjusted Relative Risks of Severe Maternal Morbidity for 5 Joint Chronic Hypertension and Hypertensive Disorders Developing During Pregnancy Subgroups, Stratified by Parity **eTable 9.** Models 1-4: Unadjusted and Adjusted Relative Risks of Severe Maternal Morbidity Without Blood Transfusion Indicator for 2 Independent Variables for Hypertensive Disorders: Chronic Hypertension and Hypertensive Disorders Developing During Pregnancy **eTable 10.** Model 4: Fully Adjusted Relative Risks of Severe Maternal Morbidity Without Blood Transfusion Indicator for 2 Independent Variables for Hypertensive Disorders: Chronic Hypertension and Hypertensive Disorders Developing During Pregnancy With Covariates. **eTable 11.** Models 1C and 2C: Unadjusted and Adjusted Relative Risks of Severe Maternal Morbidity Without Blood Transfusion Indicator for 5 Joint Chronic Hypertension and Hypertensive Disorders Developing During Pregnancy Subgroups **eTable 12.** Model 2C: Fully Adjusted Relative Risks of Severe Maternal Morbidity Without Blood Transfusion Indicator for 5 Joint Chronic Hypertension and Hypertensive Disorders Developing During Pregnancy Subgroups With Covariates **eFigure 1.** Selection Criteria to Identify 1 Singleton Live Birth or Stillbirth Per Individual From 2009 to 2019 **eFigure 2.** Directed Acyclic Graph of Causal and Noncausal Paths **eFigure 3.** Risk Differences for Severe Maternal Morbidity Rates Minus Rates for the Respective Reference Groups

Supplement 2.
Data Sharing Statement
