# Supplementary material for: Severe Maternal Morbidity Associated With Chronic Hypertension, Preeclampsia, and Gestational Hypertension
Source: JAMA Netw Open. 2025 Jan 28;8(1):e2451406. doi: 10.1001/jamanetworkopen.2024.51406 (PMC11775729; doi:10.1001/jamanetworkopen.2024.51406)
Supplement: Supplement 1. — eTable 1. Severe Maternal Morbidity Rates at Delivery Hospitalization Overall and for the 21 Individual Indicators by Chronic Hypertension Status and Stratified by Hypertensive Disorders Developing During Pregnancy eTable 2. Definition of Severe Maternal Morbidity Indicators. eTable 3. Maternal Characteristics by Chronic Hypertension Status and Hypertensive Disorders Developing During Pregnancy eTable 4. Gestational Age at Delivery, Prevalence of Preterm Deliveries, and Mode of Delivery According to Chronic Hypertension and Hypertensive Disorders Developing During Pregnancy eTable 5. Model 4: Fully Adjusted Relative Risks of Severe Maternal Morbidity for 2 Independent Variables for Hypertensive Disorders: Chronic Hypertension and Hypertensive Disorders Developing During Pregnancy With Covariates eTable 6. Causal Mediation Analysis eTable 7. Model 2C: Fully Adjusted Relative Risks of Severe Maternal Morbidity for 5 Joint Chronic Hypertension and Hypertensive Disorders Developing During Pregnancy Subgroups With Covariates eTable 8. Models 2C.1 and 2C.2: Fully Adjusted Relative Risks of Severe Maternal Morbidity for 5 Joint Chronic Hypertension and Hypertensive Disorders Developing During Pregnancy Subgroups, Stratified by Parity eTable 9. Models 1-4: Unadjusted and Adjusted Relative Risks of Severe Maternal Morbidity Without Blood Transfusion Indicator for 2 Independent Variables for Hypertensive Disorders: Chronic Hypertension and Hypertensive Disorders Developing During Pregnancy eTable 10. Model 4: Fully Adjusted Relative Risks of Severe Maternal Morbidity Without Blood Transfusion Indicator for 2 Independent Variables for Hypertensive Disorders: Chronic Hypertension and Hypertensive Disorders Developing During Pregnancy With Covariates. eTable 11. Models 1C and 2C: Unadjusted and Adjusted Relative Risks of Severe Maternal Morbidity Without Blood Transfusion Indicator for 5 Joint Chronic Hypertension and Hypertensive Disorders Developing During Pregnancy Subgroups [file jamanetwopen-e2451406-s001.pdf]

Supplemental Online Content

Gunderson EP, Greenberg M, Najem M, et al. Severe Maternal Morbidity Associated with Chronic hHypertension, Preeclampsia, and Gestational Hypertension. *JAMA Netw Open*. 2024;7(12):e2451406. doi:10.1001/jamanetworkopen.2024.51406

eTable 1. Severe Maternal Morbidity Rates at Delivery Hospitalization Overall and for the 21 Individual Indicators by Chronic Hypertension Status and Stratified by Hypertensive Disorders Developing During Pregnancy

eTable 2. Definition of Severe Maternal Morbidity Indicators.

eTable 3. Maternal Characteristics by Chronic Hypertension Status and Hypertensive Disorders Developing During Pregnancy.

eTable 4. Gestational Age at Delivery, Prevalence of Preterm Deliveries, and Mode of Delivery According to Chronic Hypertension and Hypertensive Disorders Developing During Pregnancy

eTable 5. Model 4: Fully Adjusted Relative Risks of Severe Maternal Morbidity for 2 Independent Variables for Hypertensive Disorders: Chronic Hypertension and Hypertensive Disorders Developing During Pregnancy With Covariates

eTable 6. Causal Mediation Analysis

eTable 7. Model 2C: Fully Adjusted Relative Risks of Severe Maternal Morbidity for 5 Joint Chronic Hypertension and Hypertensive Disorders Developing During Pregnancy Subgroups With Covariates

eTable 8. Models 2C.1 and 2C.2: Fully Adjusted Relative Risks of Severe Maternal Morbidity for 5 Joint Chronic Hypertension and Hypertensive Disorders Developing During Pregnancy Subgroups, Stratified by Parity

eTable 9. Models 1-4: Unadjusted and Adjusted Relative Risks of Severe Maternal Morbidity Without Blood Transfusion Indicator for 2 Independent Variables for Hypertensive Disorders: Chronic Hypertension and Hypertensive Disorders Developing During Pregnancy

eTable 10. Model 4: Fully Adjusted Relative Risks of Severe Maternal Morbidity Without Blood Transfusion Indicator for 2 Independent Variables for Hypertensive Disorders: Chronic Hypertension and Hypertensive Disorders Developing During Pregnancy With Covariates.

eTable 11. Models 1C and 2C: Unadjusted and Adjusted Relative Risks of Severe Maternal Morbidity Without Blood Transfusion Indicator for 5 Joint Chronic Hypertension and Hypertensive Disorders Developing During Pregnancy Subgroups

eTable 12. Model 2C: Fully Adjusted Relative Risks of Severe Maternal Morbidity Without Blood Transfusion Indicator for 5 Joint Chronic Hypertension and Hypertensive Disorders Developing During Pregnancy Subgroups With Covariates

© 2024 Gunderson EP et al. *JAMA Network Open.*

eFigure 1. Selection Criteria to Identify 1 Singleton Live Birth or Stillbirth Per Individual From 2009 to 2019

eFigure 2. Directed Acyclic Graph of Causal and Noncausal Paths

eFigure 3. Risk Differences for Severe Maternal Morbidity Rates Minus Rates for the Respective Reference Groups

This supplemental material has been provided by the authors to give readers additional information about their work.

© 2024 Gunderson EP et al. *JAMA Network Open.*

eTable1. Severe Maternal Morbidity Rates at Delivery Hospitalization Overall and for the 21 Individual Indicators by Chronic Hypertension Status and Stratified by Hypertensive Disorders Developing During Pregnancy

**CDC Severe Maternal Morbidity Rates (95% CI)<br>21 Individual CDC Indicators<br>(Rates: n per 10,000 women)**	Chronic Hypertension			No Chronic Hypertension			
	ALL	Superimposed preeclampsia	No preeclampsia	ALL	Preeclampsia	Gestational hypertension	No preeclampsia or gestational hypertension
	N=13,626	N=4,297	N=9,329	N=249,892	N=11,774	N=11,514	N=226,604
Severe maternal morbidity (Overall)	568 (416.9)	386 (898.3)	182 (195.1)	5218 (208.8)	1100 (934.3)	360 (312.7)	3758 (165.8)
95% Confidence Intervals (CI)	383.9-451.8	814.5-987.8	168.0-225.2	203.2-214.5	882.3-988.3	281.6-346.1	160.6-171.2
**Rates (per 10,000 births) for 21 CDC indicators:**							
1. Acute myocardial infarction	1 (0.7)	1 (2.3)	0 (0.0)	7 (0.3)	3 (2.5)	0 (0.0)	4 (0.2)
2. Aneurysm	3 (2.2)	1 (2.3)	2 (2.1)	6 (0.2)	0 (0.0)	1 (0.9)	5 (0.2)
3. Acute renal failure	74 (54.3)	63 (146.6)	11 (11.8)	395 (15.8)	162 (137.6)	48 (41.7)	185 (8.2)
4. Adult respiratory distress syndrome	25 (18.3)	20 (46.5)	5 (5.4)	135 (5.4)	33 (28.0)	12 (10.4)	90 (4.0)
5. Amniotic fluid embolism	3 (2.2)	2 (4.7)	1 (1.1)	21 (0.8)	1 (0.8)	3 (2.6)	17 (0.8)
6. Cardiac arrest/ventricular fibrillation	3 (2.2)	2 (4.7)	1 (1.1)	19 (0.8)	3 (2.5)	3 (2.6)	13 (0.6)
7. Conversion of cardiac rhythm	3 (2.2)	2 (4.7)	1 (1.1)	12 (0.5)	2 (1.7)	2 (1.7)	8 (0.4)
8. Disseminated intravascular coagulation	51 (37.4)	28 (65.2)	23 (24.7)	1,002 (40.1)	138 (117.2)	54 (46.9)	810 (35.7)
9. Eclampsia	136 (99.8)	135 (314.2)	---	308 (12.3)	305 (259.0)	---	---
10. Heart failure/arrest during surgery or procedure	2 (1.5)	1 (2.3)	1 (1.1)	15 (0.6)	2 (1.7)	0 (0.0)	13 (0.6)
11. Puerperal Stroke or Other cerebrovascular disorders	16 (11.7)	11 (25.6)	5 (5.4)	97 (3.9)	16 (13.6)	9 (7.8)	72 (3.2)
11a. Ischemic stroke	7 (5.1)	3 (7.0)	4 (4.3)	44 (1.8)	11 (9.3)	4 (3.5)	29 (1.3)
11b. Intracerebral hemorrhage	2 (1.5)	2 (4.7)	0 (0.0)	6 (0.2)	2 (1.7)	0 (0.0)	4 (0.2)
11c. Subarachnoid hemorrhage	2 (1.5)	2 (4.7)	0 (0.0)	4 (0.2)	2 (1.7)	1 (0.9)	1 (0.0)
11d. Other cerebrovascular disorders	8 (5.9)	7 (16.3)	1 (1.1)	55 (2.2)	6 (5.1)	5 (4.3)	44 (1.9)
12. Pulmonary edema / Acute heart failure	43 (31.6)	33 (76.8)	10 (10.7)	164 (6.6)	45 (38.2)	17 (14.8)	102 (4.5)
13. Severe anesthesia complications	5 (3.7)	0 (0.0)	5 (5.4)	27 (1.1)	3 (2.5)	2 (1.7)	22 (1.0)
14. Sepsis	40 (29.4)	27 (62.8)	13 (13.9)	781 (31.3)	95 (80.7)	77 (66.9)	609 (26.9)
15. Shock	7 (5.1)	5 (11.6)	2 (2.1)	102 (4.1)	17 (14.4)	9 (7.8)	76 (3.4)
16. Sickle cell disease with crisis	1 (0.7)	0 (0.0)	1 (1.1)	6 (0.2)	4 (3.4)	0 (0.0)	2 (0.1)
17. Air and thrombotic embolism	15 (11.0)	9 (20.9)	6 (6.4)	106 (4.2)	6 (5.1)	4 (3.5)	96 (4.2)
18. Blood products transfusion	262 (192.3)	145 (337.4)	117 (125.4)	2,759(110.4)	487 (413.6)	193 (167.6)	2,079 (91.7)
19. Hysterectomy	23 (16.9)	8 (18.6)	15 (16.1)	202 (8.1)	10 (8.5)	8 (6.9)	184 (8.1)
20. Temporary tracheostomy	0	0	0	5 (0.2)	2 (1.7)	2 (1.7)	1 (0.0)
21. Ventilation	19 (13.9)	12 (27.9)	7 (7.5)	56 (2.2)	9 (7.6)	7 (6.1)	40 (1.8)

© 2024 Gunderson EP et al. *JAMA Network Open.*

eTable2. Definition of Severe Maternal Morbidity (SMM) Indicators based on Diagnosis and Procedure Codes

SMM Indicators	ICD9 diagnosis codes	ICD10 diagnosis codes
**Acute myocardial infarction**	substr(DX, 1, 3)='410'	DX IN ('I21.01', 'I21.02', 'I21.09', 'I21.11', 'I21.19', 'I21.21', 'I21.29', 'I21.3', 'I21.4', 'I21.9', 'I21.A1', 'I21.A9', 'I22.0', 'I22.1', 'I22.2', 'I22.8', 'I22.9')
**Aneurysm**	substr(DX, 1, 3)='441'	DX IN ('I71.00', 'I71.01', 'I71.02', 'I71.03', 'I71.1', 'I71.2', 'I71.3', 'I71.4', 'I71.5', 'I71.6', 'I71.8', 'I71.9', 'I79.0')
**Acute renal failure**	substr(dx, 1, 5)='669.3' or dx in('584.5', '584.6', '584.7', '584.8','584.9')	dx in ('N17.0', 'N17.1', 'N17.2', 'N17.8', 'N17.9', 'O90.4')
**Acute respiratory distress syndrome**	substr(dx, 1, 5)='518.5' or dx in('518.81', '518.82', '518.84', '799.1')	Dx in ('J80', 'J95.1', 'J95.2', 'J95.3', 'J95.821', 'J95.822', 'J96.00', 'J96.01', 'J96.02', 'J96.20', 'J96.21', 'J96.22', 'R09.2')
**Amniotic fluid embolism**	substr(DX, 1, 5)='673.1'	DX IN ('O88.12', 'O88.13') or substr(DX, 1,6) ='O88.11'
**Cardiac arrest**	dx in('427.41', '427.42', '427.5')	DX IN ('I46.2', 'I46.8', 'I46.9', 'I49.01', 'I49.02')
**Disseminated intravascular coagulation**	substr(DX, 1, 5)='666.3' or DX in('286.6', '286.9')	DX IN ('D65', 'D68.8', 'D68.9', 'O72.3')
**Eclampsia**	substr(DX,1,5)='642.6'	DX IN ('O15.00', 'O15.02', 'O15.03', 'O15.1', 'O15.2', 'O15.9')

© 2024 Gunderson EP et al. *JAMA Network Open.*

SMM Indicators	ICD9 diagnosis codes	ICD10 diagnosis codes
**Heart failure**	DX in('997.1')	DX IN ('I97.120', 'I97.121', 'I97.130', 'I97.131', 'I97.710', 'I97.711')
**Stroke and Cerebrovascular**		
**Intracerebral hemorrhage**	DX='431'	DX IN ('I61.0', 'I61.1', 'I61.2', 'I61.3', 'I61.4', 'I61.5', 'I61.6', 'I61.8', 'I61.9')
**Ischemic stroke**	DX IN ('433.01', '433.11', '433.21', '433.31', '433.81', '433.91', '434.01', '434.11', '434.91', '437.1')	DX IN ('I63.00', 'I63.011', 'I63.012', 'I63.013', 'I63.019', 'I63.02', 'I63.031', 'I63.032', 'I63.033', 'I63.039', 'I63.09',<br>'I63.10', 'I63.111', 'I63.112', 'I63.113', 'I63.119', 'I63.12', 'I63.131', 'I63.132', 'I63.133', 'I63.139', 'I63.19', 'I63.20', 'I63.211', 'I63.212', 'I63.213', 'I63.219', 'I63.22', 'I63.231', 'I63.232', 'I63.233', 'I63.239', 'I63.29', 'I63.30', 'I63.311', 'I63.312', 'I63.313', 'I63.319', 'I63.321', 'I63.322', 'I63.323', 'I63.329', 'I63.331', 'I63.332', 'I63.333', 'I63.339', 'I63.341', 'I63.342', 'I63.343', 'I63.349', 'I63.39', 'I63.40', 'I63.411', 'I63.412', 'I63.413', 'I63.419', 'I63.421', 'I63.422', 'I63.423', 'I63.429', 'I63.431', 'I63.432', 'I63.433', 'I63.439', 'I63.441', 'I63.442', 'I63.443', 'I63.449', 'I63.49', 'I63.50', 'I63.511', 'I63.512', 'I63.513', 'I63.519', 'I63.521', 'I63.522', 'I63.523', 'I63.529', 'I63.531', 'I63.532', 'I63.533', 'I63.539','I63.541', 'I63.542', 'I63.543', 'I63.549', 'I63.59', 'I63.6', 'I63.8', 'I63.9', 'I67.82')
**Subarachnoid hemorrhage**	Dx in(‘430’)	DX IN ('I60.00','I60.01','I60.02'<br>,'I60.10','I60.11','I60.12','I60.2','I60.30','I60.31', 'I60.32',<br>'I60.4','I60.50','I60.51'<br>, 'I60.52', 'I60.6','I60.7', 'I60.8', 'I60.9')
**Other cerebrovascular disorders**	DX in('997.02')<br>or substr(DX,1, 3)='436'<br>or substr(DX, 1, 5) in ('437.0' ,’437.2,<br>‘437.3’,’437.4’,’437.5’,’437.6’,’437.7’,437.8,’437.9’)<br>or substr(DX, 1, 5)='671.5'<br>or substr(DX, 1, 5)='674.0'	DX IN ('I62.1', 'I62.9', 'I65.1', 'I65.8', 'I65.9', 'I66.3', 'I66.8', 'I66.9', 'I67.0', 'I67.1', 'I67.2', 'I67.3', 'I67.4', 'I67.5', 'I67.6', 'I67.7', 'I67.9', 'I68.0', 'I68.2', 'I68.8', 'O22.51', 'O22.52', 'O22.53', 'I97.810', 'I97.811', 'I97.820', 'I97.821', 'O87.3') or substr(DX, 1, 5)='I65.0' or substr(DX, 1, 5)='I65.2' or substr(DX, 1,5)='I66.0' or<br>substr(DX, 1, 5)='I66.1' or<br>substr(DX, 1,5)='I66.2' or<br>substr(DX, 1, 5)='674.0'

© 2024 Gunderson EP et al. *JAMA Network Open.*

SMM Indicators	ICD9 diagnosis codes	ICD10 diagnosis codes
**Pulmonary edema**	DX in('518.4', '428.1', '428.0', '428.21', '428.23', '428.31', '428.33', '428.41', '428.43')	DX IN ('J81.0', 'I50.1', 'I50.20', 'I50.21', 'I50.23', 'I50.30', 'I50.31', 'I50.33', 'I50.40', 'I50.41', 'I50.43', 'I50.9')
**Severe anesthesia complications**	substr(DX, 1, 5)='668.0' or substr(DX, 1, 5) ='668.1' or substr(DX, 1, 5)='668.2'	DX IN ('O74.0', 'O74.1', 'O74.2', 'O74.3', 'O89.01', 'O89.09', 'O89.1', 'O89.2')
**Sepsis**	DX in('995.91','995.92') or substr(DX, 1,3) ='038' or substr(DX, 1, 5) ='670.2'	DX IN (‘O85’,’O86.04’, ‘T80.211A’,T81.4XXA’,’T81.44’, ‘T81.44XA’,T81.44XD’, ‘T81.44XS’,’R65.20’, ‘A40.0’, ‘A40.1’, ‘A40.3’, ‘A40.8’, ‘A40.9’, ‘A41.01’, ‘A41.02’, ‘A41.1’, ‘A41.2’, ‘A41.3’, ‘A41.4’, ‘A41.50’, ‘A41.51’, ‘A41.52’, ‘A41.53’, ‘A41.59’, ‘A41.81’, ‘A41.89’, ‘A41.9’, ‘A32.7’
**Shock**	substr(DX, 1, 5)='669.1' or substr(DX, 1, 5)='785.5' or substr(DX, 1, 5) ='998.0' or (DX in ('995.0', '995.4')	DX IN ('O75.1', 'R57.0', 'R57.1', 'R57.8', 'R57.9', 'R65.21', 'T78.2XXA', 'T88.2XXA', 'T88.6XXA', 'T81.10XA', 'T81.11XA', 'T81.19XA')
**Sickle cell disease with crisis**	DX in('282.42', '282.62', '282.64', '282.69')	DX IN ('D57.00', 'D57.01', 'D57.02', 'D57.211', 'D57.212', 'D57.219', 'D57.411', 'D57.412', 'D57.419', 'D57.811', 'D57.812', 'D57.819')
**AIR and thrombolytic embolism**	substr(dx, 1, 5)='415.1' or substr(dx,1,5) ='673.0' or substr(DX, 1, 5) ='673.2' or substr(DX, 1, 5) ='673.3' or substr(DX1,5)='673.8'	dx in ('I26.0', 'I26.01', 'I26.02', 'I26.09', 'I26.90', 'I26.92', 'I26.99','O88.011', 'O88.012', 'O88.013', 'O88.014', 'O88.015', 'O88.016', 'O88.017', 'O88.018', 'O88.019', 'O88.02', 'O88.03', 'O88.211', 'O88.212', 'O88.213', 'O88.214', 'O88.215', 'O88.216', 'O88.217', 'O88.218', 'O88.219', 'O88.22', 'O88.23', 'O88.311', 'O88.312', 'O88.313', 'O88.314', 'O88.315', 'O88.316', 'O88.317', 'O88.318', 'O88.319', 'O88.32', 'O88.33', 'O88.81', 'O88.82', 'O88.83')
**Embolism**	DX IN ('673.00', '673.01', '673.02', '673.03', '673.04', '673.10', '673.11', '673.12', '673.13', '673.14', '673.30', '673.31', '673.32', '673.33', '673.34', '673.20', '673.21', '673.22', '673.23', '673.24', '673.80', '673.81', '673.82', '673.83', '673.84')	DX IN ('O88.011', 'O88.012', 'O88.013', 'O88.019', 'O88.02', 'O88.03', 'O88.111', 'O88.112', 'O88.113', 'O88.119', 'O88.12', 'O88.13', 'O88.311', 'O88.312', 'O88.313', 'O88.319', 'O88.32', 'O88.33', 'O88.811', 'O88.812', 'O88.813', 'O88.819', 'O88.82', 'O88.83', 'I26.01', 'I26.02', 'I26.09', 'I26.90', 'I26.92', 'I26.99', 'O88.211', 'O88.212', 'O88.213', 'O88.219', 'O88.22', 'O88.23')

© 2024 Gunderson EP et al. *JAMA Network Open.*

SMM indicator	Procedure codes	Local procedure codes
**Blood products transfusion**	(substr(PX,**1**,**4**)='99.0'<br>AND PX_CODETYPE = '09')<br>or PX IN ('30233H1','30233L1', '30233K1',<br>'30233M1', '30233N1','30233P1','30233R1','30233T1',<br>'30233H0','30233L0', '30233K0',<br>'30233M0', '30233N0','30233P0','30233R0','30233T0',<br>'30230H1','30230L1', '30230K1',<br>'30230M1', '30230N1','30230P1','30230R1','30230T1',<br>'30230H0','30230L0', '30230K0',<br>'30230M0', '30230N0','30230P0','30230R0','30230T0',<br>'30240H1','30240L1', '30240K1',<br>'30240M1', '30240N1','30240P1','30240R1','30240T1',<br>'30240H0','30240L0', '30240K0',<br>'30240M0', '30240N0','30240P0','30240R0','30240T0',<br>'30243H1','30243L1', '30243K1',<br>'30243M1', '30243N1','30243P1','30243R1','30243T1',<br>'30243H0','30243L0', '30243K0',<br>'30243M0', '30243N0','30243P0','30243R0','30243T0',<br>'30250H1','30250L1', '30250K1',<br>'30250M1', '30250N1','30250P1','30250R1','30250T1',<br>'30250H0','30250L0', '30250K0',<br>'30250M0', '30250N0','30250P0','30250R0','30250T0',<br>'30253H1','30253L1', '30253K1',<br>'30253M1', '30253N1','30253P1','30253R1','30253T1',<br>'30253H0','30253L0', '30253K0',<br>'30253M0', '30253N0','30253P0','30253R0','30253T0',<br>'30260H1','30260L1', '30260K1',<br>'30260M1', '30260N1','30260P1','30260R1','30260T1',<br>'30260H0','30260L0', '30260K0',<br>'30260M0', '30260N0','30260P0','30260R0','30260T0',<br>'30263H1','30263L1', '30263K1',<br>'30263M1', '30263N1','30263P1','30263R1','30263T1',<br>'30263H0','30263L0', '30263K0',<br>'30263M0', '30263N0','30263P0','30263R0','30263T0')<br>AND PX_CODETYPE = '10')	

© 2024 Gunderson EP et al. *JAMA Network Open.*

SMM indicator	Procedure codes	Local procedure codes
**Cardiac Rhythm**	substr(b.px,1,3)='99.6'<br>AND B.PX_<br>CODETYPE = '09')<br>OR (b.PX IN ('5A2204Z',<br>'5A12012') AND B.PX_CODETYPE = '10')	
**Hysterectomy**	px like '68.3%' or px like '68.4%' or px like '68.5%' or px like '68.6%' or px like '68.7%' or px like '68.8%' or px like '68.9%' AND PX_CODETYPE='09'<br>OR (PX IN ('0UT90ZZ', '0UT94ZZ','0UT97ZZ', '0UT98ZZ', '0UT9FZZ') AND PX_CODETYPE = '10')	PX IN '0UT90ZL','0UT94ZL' ,'0U590ZZ','0U593ZZ', '0U594ZZ','0U597ZZ', '0U598ZZ') AND PX_CODETYPE='10')<br>OR (PX IN ('51925', '58150', '58152', '58180', '58200', '58210', '58240','58260', '58262', '58263', '58267', '58270', '58275', '58280', '58285', '58290', '58291', '58292', '58293', '58294', '58541', '58542', '58543',<br>'58544', '58548', '58550', '58552', '58553', '58554', '58570', '58571', '58572', '58573', '58575', '58953', '58954', '58956') AND PX_CODETYPE='C4')<br>OR (PX IN('106622', '106623', '106624', '106625', '106626', '129189', '132481', '170364', '170365', '170437', '170447', '170448', '189452','189453', '189456', '189891', '192289', '192290', '192291', '192292',<br>'207781', '207872', '211512', '220000', '221026', '221027', '221028','222537', '222553', '223334', '223341', '223342', '223343', '223344','223345', '223346', '223347', '223348', '225914', '226750', '226751',<br>'226752', '226753', '226754', '226755', '226756', '226757', '226980', '227863', '227864', '227866', '227867', '229369', '230673', '232087','232104', '237645', '240485', '243868', '245208', '245725', '245726',<br>'246158', '246159', '246161', '246412', '248854', '248855', '248856', '248857', '248858', '257956', '267763', '283127', '283128', '346379','371473', '591558', '650239', '678277', '678278', '678279', '678280',<br>'678281') AND PX_CODETYPE='LO') OR PX='S2078')
**Temporary tracheostomy**	PX='31.1' AND PX_CODETYPE='09')<br>OR (PX IN ('0B110Z4', '0B110F4', '0B113Z4', '0B113F4', '0B114Z4', '0B114F4') AND PX_CODETYPE='10')	PX in ('106423','138945','192674','207941',<br>'220300','222582','223996',<br>'225011','225235','257285',<br>'31.21','31600','31601','31603',<br>'31605','31610','851500')

© 2024 Gunderson EP et al. *JAMA Network Open.*

SMM indicator	Procedure codes	Local procedure codes
**Ventilation**	PX in ('93.90', '96.01', '96.02', '96.03', '96.05') AND PX_CODETYPE='09') OR (PX IN ('5A1935Z', '5A1945Z', '5A1955Z') AND PX_CODETYPE='10')	PX in ('218506','218505','224598','244487', '106818')

© 2024 Gunderson EP et al. *JAMA Network Open.*

eTable3. Maternal Characteristics by Chronic Hypertension Status and Hypertensive Disorders Developing During Pregnancy; Total n=263,518 women with a singleton birth (2009-2019).

Chronic Hypertension and Hypertensive Disorders Developing During Pregnancy Subgroups	Chronic Hypertension		No Chronic Hypertension		
Maternal Characteristics n (col%) or Mean (SD)	Superimposed preeclampsia n=4,297	No preeclampsia n=9,329	Preeclampsia n=11,774	Gestational hypertension n=11,514	No preeclampsia or gestational hypertension n=226,604
Maternal age, y,	33.0 (5.49)	33.0 (5.25)	30.7 (5.76)	31.1 (5.41)	30.9 (5.27)
Age categories, n (%)					
18-25 y	487 (11.3)	882 (9.5)	2,631 (22.3)	2,105 (18.3)	41,016 (18.1)
26-30 y	1,006 (23.4)	2,381 (25.5)	3,368 (28.6)	3,493 (30.3)	70,975 (31.3)
31-35 y	1,483 (34.5)	3,257 (34.9)	3,598 (30.6)	3,789 (32.9)	75,954 (33.5)
36-40 y	1,011 (23.5)	2,240 (24.0)	1,793 (15.2)	1,741 (15.1)	32,932 (14.5)
41-45 y	310 (7.2)	569 (6.1)	384 (3.3)	386 (3.4)	5727 (2.5)
Racial and Ethnic groups, n (%)					
Asian	989 (23.0)	1,691 (18.1)	2,645 (22.5)	2,222 (19.3)	58,543 (25.8)
American Indian/Native Alaskan	10 (0.2)	33 (0.4)	33 (0.3)	35 (0.3)	712 (0.3)
Black	618 (14.4)	1,301 (13.9)	1,155 (9.8)	972 (8.4)	15,939 (7.0)
Hispanic	1,139 (26.5)	2,155 (23.1)	3,401 (28.9)	2,479 (21.5)	60,123 (26.5)
Mixed/Not specified	123 (2.9)	301 (3.2)	371 (3.2)	407 (3.5)	7,681 (3.4)
Native Hawaiian/Pacific Islander	57 (1.3)	70 (0.8)	94 (0.8)	106 (0.9)	1,645 (0.7)
White	1,361 (31.7)	3,778 (40.5)	4,075 (34.6)	5,293 (46.0)	81,961 (36.2)
Prenatal parity, n (%)					
Nulliparous	2,571 (59.8)	4,506 (48.3)	9,345 (79.4)	8,678 (75.4)	124,849 (55.1)
Primiparous	926 (21.5)	2,656 (28.5)	1,544 (13.1)	1,809 (15.7)	63,841 (28.2)
Biparous	511 (11.9)	1,273 (13.6)	609 (5.2)	705 (6.1)	25,911 (11.4)
Multiparous (3 or more births)	289 (6.7)	894 (9.6)	276 (2.3)	322 (2.8)	12,003 (5.3)
Diabetes status, n (%)					
Pregestational diabetes	353 (8.2)	559 (6.0)	232 (2.0)	124 (1.1)	1,202 (0.5)
Gestational diabetes	1,079 (25.1)	2,111 (22.6)	1,807 (15.3)	1,608 (14.0)	25,102 (11.1)
None	2,865 (66.7)	6,659 (71.4)	9,735 (82.7)	9,782 (85.0)	200,300 (88.4)
Pre-pregnancy weight, kg,	86.3 (24.0)	85.9 (23.8)	73.1 (18.9)	76.6 (19.8)	68.2 (16.1)
Height, cm,	162.6 (7.3)	163.8 (7.3)	161.8 (7.0)	163.6 (7.2)	162.5 (6.99)
Pre-pregnancy BMI, kg/m^2,	32.5 (8.03)	31.9 (8.2)	27.8 (6.6)	28.6 (6.9)	25.8 (5.7)
Pre-pregnancy BMI, n (%)					
Underweight (<18.5)	16 (0.4)	43 (0.5)	222 (1.9)	140 (1.2)	6,399 (2.8)
Normal weight (18.5-24.9)	751 (17.6)	1,961 (21.1)	4,504 (38.5)	4,008 (35.0)	114,686 (51.0)
Overweight (25-29.9)	1,065 (24.9)	2,352 (25.3)	3,316 (28.3)	3,315 (28.9)	60,600 (26.9)
Obesity class I (30-34.9)	1,021 (23.9)	2,013 (21.7)	1,993 (17.0)	2,090 (18.2)	26,481 (11.8)
Obesity class II (35-39.9)	688 (16.1)	1,424 (15.3)	1,011 (8.6)	1,095 (9.6)	10,776 (4.8)
Obesity class III (≥40)	731 (17.1)	1,502 (16.2)	660 (5.6)	807 (7.0)	5,968 (2.7)
Unknown	25 (0.6)	34 (0.4)	68 (0.6)	59 (0.5)	1,694 (0.7)
Gestational age at first prenatal care visit, weeks	7.9 (2.0)	7.9 (2.0)	8.0 (2.0)	8.0 (1.9)	8.2 (2.0)
Gestational age at delivery, weeks	36.8 (3.0)	38.6 (2.5)	38.2 (2.6)	39.3 (1.6)	39.3 (1.96)
Total Gestational Weight Gain, kg	12.3 (8.0)	11.4 (8.0)	15.0 (7.2)	14.7 (7.4)	13.4 (6.3)

© 2024 Gunderson EP et al. *JAMA Network Open.*

eTable3. Maternal Characteristics, Continued					
Chronic Hypertension and Hypertensive Disorders Developing During Pregnancy Subgroups	Chronic Hypertension		No Chronic Hypertension		
Maternal Characteristics n (col%) or Mean (SD)	Superimposed preeclampsia n=4,297	No preeclampsia n=9,329	Preeclampsia n=11,774	Gestational hypertension n=11,514	No preeclampsia or gestational hypertension n=226,604
Social Factors					
Neighborhood deprivation index (NDI), n (%)					
≤ -1 (least deprived)	365 (8.5)	833 (8.9)	1,192 (10.1)	1,333 (11.6)	26,198 (11.6)
>-1 and ≤0	2,119 (49.4)	4,601 (49.4)	5,828 (49.6)	5,906 (51.4)	114,213(50.5)
>0 and ≤1	1,206 (28.1)	2,600 (27.9)	3,181 (27.1)	2,986 (26.0)	58,575 (25.9)
>1 (most deprived)	598 (13.9)	1,279 (13.7)	1,553 (13.2)	1,274 (11.1)	27,249 (12.0)
Unknown	9 (0.2)	16 (0.2)	20 (0.2)	15 (0.1)	369 (0.2)
Type of health insurance, n (%)					
Government: MediCal, Medicaid, State-subsidized	378 (8.8)	845 (9.1)	930 (7.9)	846 (7.3)	16,625 (7.3)
Employer/Self-funded/Other	3,919 (91.2)	8,484 (90.9)	10,844 (92.1)	10,668(92.-)	209,979(92.7)
Lifestyle Behaviors					
Smoking status, n (%)					
Current	329 (7.7)	723 (7.8)	689 (5.9)	799 (6.9)	12,599 (5.6)
Former	683 (15.9)	1,444 (15.5)	1,590 (13.5)	1,725 (15.0)	27,094 (12.0)
Never	3,260 (75.9)	7,117 (76.3)	9,421 (80.0)	8,927 (77.5)	185,821(82.0)
Unknown	25 (0.6)	45 (0.5)	74 (0.6)	63 (0.5)	1090 (0.5)

© 2024 Gunderson EP et al. *JAMA Network Open.*

eTable4. Gestational Age at Delivery, Prevalence of Preterm Deliveries and Mode of Delivery According to Chronic Hypertension and Hypertensive Disorders Developing During Pregnancy

Chronic Hypertension and Hypertensive Disorders Developing During Pregnancy	All	Chronic Hypertension N= 13,626		No Chronic Hypertension N=249,892		
		Superimposed preeclampsia	No preeclampsia	Preeclampsia	Gestational hypertension	No preeclampsia or gestational hypertension
	N=263,518	n=4,297 (31.5%)	n=9,329	n=11,774 (4.7%)	n=11,514 (4.6%)	n=226,604
Gestational age at delivery, weeks, mean (SD)	39.2 (2.1)	36.8 (3.0)	38.6 (2.5)	38.2 (2.6)	39.3 (1.6)	39.3 (2.0)
Gestational age categories, n (%)						
Early preterm	5,172 (2.0)	570 (13.3)	330 (3.5)	707 (6.0)	102 (0.9)	3,463 (1.5)
Late preterm 34–36 6/7 weeks	13,560 (5.1)	1,048 (24.4)	626 (6.7)	1,657 (14.1)	469 (4.1)	9,760 (4.3)
37 to 40 weeks	160,410 (60.9)	2,412 (56.1)	6,639 (71.2)	6,789 (57.7)	7,044 (61.2)	137,526 (60.7)
>40 weeks	84,376 (32.0)	267 (6.2)	1,734 (18.6)	2,621 (22.3)	3,899 (33.9)	75,855 (33.5)
Mode of delivery, n (%)*						
Normal spontaneous	176,406 (66.9)	1,904 (44.3)	5,315 (57.0)	6,239 (53.0)	7,370 (64.0)	155,578 (68.7)
Assisted vaginal delivery	15,532 (5.9)	207 (4.8)	419 (4.5)	846 (7.2)	746 (6.5)	13,314 (5.9)
C-section	69,550 (26.4)	2,156 (50.2)	3,480 (37.3)	4,592 (39.0)	3,342 (29.0)	55,980 (24.7)
Unknown	2,030 (0.8)	30 (0.7)	115 (1.2)	97 (0.8)	56 (0.5)	1,732 (0.8)

© 2024 Gunderson EP et al. *JAMA Network Open.*

eTable5. Model 4: Fully Adjusted Relative Risks (95% Confidence Intervals) of Severe Maternal Morbidity for 2 Independent Variables for Hypertensive Disorders: Chronic Hypertension and Hypertensive Disorders Developing During Pregnancy with Covariates.

**Entire Cohort (n=263,518)**	**Adjusted Relative Risk of SMM**	
**Model 4 = Fully Adjusted with Covariates**	**aRR (95% CI)**	**n/N (%)**
1) Chronic hypertension status:		
Chronic hypertension	1.04 (0.94-1.14)	568/13,626 (4.2)
No chronic hypertension	1.0 (Referent)	5,218/249,892 (2.1)
2) Hypertensive disorders developing during pregnancy:		
Any preeclampsia (with or without chronic hypertension)	5.00 (4.69-5.34)	1,486/16,071 (9.2)
Gestational hypertension[a]	1.77 (1.59-1.98)	360/11,514 (3.1)
No preeclampsia or gestational hypertension (with or without chronic hypertension)	1.0 (Referent)	3,940/235,933 (1.7)
Covariates:		
Racial and ethnic groups		
Asian	1.37 (1.28-1.47)	1,705/66,090 (2.6)
American Indian/Native Alaskan	0.99 (0.59-1.66)	14/823 (1.7)
Black	1.29 (1.16-1.42)	510/19,985 (2.6)
Hispanic	1.24 (1.15-1.33)	1,516/69,297 (2.2)
Mixed/Not specified	1.08 (0.93-1.26)	180/8,883 (2.0)
Native Hawaiian/Pacific Islander	1.50 (1.16-1.95)	56/1,972 (2.8)
White	1.0 (Referent)	1,805/96,468 (1.9)
Age, year	1.03 (1.02-1.03)	
Parity		
Nulliparous (no prior births)	1.60 (1.50-1.69)	3,993/149,949 (2.7)
Parous (≥1 prior births)	1.0 (Referent)	1,793/113,569 (1.6)
Prepregnancy BMI, kg/m^2	0.99 (0.99-1.00)	-
Diabetes status		
Pregestational diabetes	1.27 (1.05-1.53)	113/2,470 (4.6)
Gestational diabetes	1.04 (0.96-1.12)	821/31,707 (2.6)
None	1.0 (Referent)	4,852/229,341 (2.1)
Neighborhood deprivation index		
≤−1 (Least deprived)	1.0 (Referent)	647/29,921 (2.2)
>−1 and ≤ 0	1.03 (0.95-1.12)	2,882/132,667 (2.2)
>0 and ≤1	1.06 (0.97-1.17)	1,498/68,548 (2.2)
>1 (Most deprived)	1.14 (1.02-1.27)	747/31,953 (2.3)
Type of health insurance		
Government: MediCal, Medicaid, State-subsidized	1.30 (1.18-1.42)	495/19,624 (2.5)
Employer/Self-funded/Other	1.0 (Referent)	5,291/243,894 (2.2)
Smoking (cigarette) habit		
Current	0.97 (0.87-1.09)	316/15,139 (2.1)
Former	0.99 (0.92-1.07)	723/32,536 (2.2)
Unknown	0.94 (0.66-1.35)	28/1,297 (2.2)
Never	1.0 (Referent)	4,719/214,546 (2.2)

[a] Gestational hypertension does not apply to the chronic hypertension group.

© 2024 Gunderson EP et al. *JAMA Network Open.*

eTable6. Causal Mediation Analysis

Percentage decompositions of total excess relative risk in causal mediation analyses without exposure-mediator interaction found that 92.4% of the total effect of chronic hypertension was attributed to mediation through preeclampsia (Natural Indirect Effect) while the remaining 7.6% was attributed to the direct pathway (Natural Direct Effect). The four-way percentage decompositions of total excess relative risk in causal mediation analyses allowing for exposure-mediator interaction found that 81.7% of the total effect was attributed to mediation but not interaction (Pure Indirect), 25.6% was attributed to the direct path without interaction nor mediation (Controlled Direct Effect), -1.20% was attributed to interaction but not mediation (Reference Interaction), and -6.07% was attributed to both mediation and interaction (Mediated Interaction).

eTable6. Causal Mediation with and without interaction: Percentage Decompositions of Total Excess Relative Risk in Causal Mediation Analyses with and without Exposure-Mediator Interaction

**Excess Relative Risk**	**Percent**	**Standard Error**	**Wald 95% Confidence Limits**		**Z**	**Pr > |Z|**
**A.** Causal mediation analyses without exposure-mediator interaction						
Natural Direct	7.58	14.53	-20.90	36.05	0.52	0.6020
Natural Indirect	92.42	14.53	63.95	120.90	6.36	<.0001
**B.** Causal mediation analyses with exposure-mediator interaction						
Natural Direct	24.35	37.16	-48.48	97.18	0.66	0.5122
Natural Indirect	75.65	37.16	2.82	148.48	2.04	0.0418
Controlled Direct	25.56	36.33	-45.64	96.75	0.70	0.4817
Reference Interaction	-1.20	1.62	-4.38	1.97	-0.74	0.4566
Mediated Interaction	-6.07	7.22	-20.23	8.09	-0.84	0.4008
Pure Indirect	81.72	40.25	2.83	160.61	2.03	0.0423

© 2024 Gunderson EP et al. *JAMA Network Open.*

eTable7. Model 2C: Fully Adjusted Relative Risks (95% Confidence Intervals) of Severe Maternal Morbidity for 5 Joint Chronic Hypertension and Hypertensive Disorders Developing During Pregnancy Subgroups with Covariates.

**Entire cohort (n=263,518)**	**Adjusted Relative Risk of SMM**	
**Model 2C = Fully Adjusted with Covariates**	**aRR (95% CI)**	**n/N (%)**
5 joint chronic hypertension and hypertensive disorders developing during pregnancy subgroups:		
Chronic hypertension with superimposed preeclampsia	4.97 (4.46-5.54)	386/4,297 (9.0)
Chronic hypertension and no preeclampsia	1.17 (1.00-1.36)	182/9,329 (2.0)
No chronic hypertension with preeclampsia	5.12 (4.79-5.48)	1,100/11,774 (9.3)
Gestational hypertension[a]	1.78 (1.60-1.99)	360/11,514 (3.1)
No chronic hypertension and no preeclampsia or gestational hypertension (reference group)	1.0 (Referent)	3,758/226,604 (1.7)
Covariates:		
Racial and ethnic groups		
Asian	1.37 (1.28-1.47)	1,705/66,090 (2.6)
American Indian/Native Alaskan	0.99 (0.59-1.66)	14/823 (1.7)
Black	1.29 (1.16-1.42)	510/19,985 (2.6)
Hispanic	1.24 (1.15-1.33)	1,516/69,297 (2.2)
Mixed/Not specified	1.09 (0.93-1.26)	180/8,883 (2.0)
Native Hawaiian/Pacific Islander	1.51 (1.17-1.95)	56/1,972 (2.8)
White	1.0 (Referent)	1,805/96,468 (1.9)
Age, y	1.03 (1.02-1.03)	
Parity		
Nulliparous (no prior births)	1.59 (1.50-1.69)	3,993/149,949 (2.7)
Parous (≥1 prior births)	1.0 (Referent)	1,793/113,569 (1.6)
Prepregnancy BMI, kg/m^2	0.99 (0.99-1.00)	-
Diabetes status		
Pregestational diabetes	1.27 (1.05-1.53)	113/2,470 (4.6)
Gestational diabetes	1.03 (0.96-1.12)	821/31,707 (2.6)
None	1.0 (Referent)	4,852/229,341 (2.1)
Neighborhood deprivation index		
≤−1 (Least deprived)	1.0 (Referent)	647/29,921 (2.2)
>−1 and ≤ 0	1.03 (0.95-1.12)	2,882/132,667 (2.2)
>0 and ≤1	1.06 (0.97-1.17)	1,498/68,548 (2.2)
>1 (Most deprived)	1.14 (1.02-1.27)	747/31,953 (2.3)
Type of health insurance		
Government: MediCal, Medicaid, State-subsidized	1.29 (1.18-1.42)	495/19,624 (2.5)
Employer/Self-funded/Other	1.0 (Referent)	5,291/243,894 (2.2)
Smoking (cigarette) habit		
Current	0.97 (0.87-1.09)	316/15,139 (2.1)
Former	0.99 (0.92-1.07)	723/32,536 (2.2)
Unknown	0.94 (0.66-1.35)	28/1,297 (2.2)
Never	1.0 (Referent)	4,719/214,546 (2.2)

[a] Gestational hypertension does not apply to the chronic hypertension group

© 2024 Gunderson EP et al. *JAMA Network Open.*

eTable8. Models 2C.1 and 2C.2: Fully Adjusted Relative Risks (95% Confidence Intervals) of Severe Maternal Morbidity for 5 Joint Chronic Hypertension and Hypertensive Disorders Developing During Pregnancy Subgroups Stratified by Parity (Primiparous vs. Multiparous).

**Models Stratified by Parity Groups**	**Adjusted Relative Risk (aRR) of SMM, and Stratified by Parity**		
**Combination of Chronic Hypertension and Pregnancy Hypertensive Groups**	**Model 2C All N=263,518**	**Model 2C.1 Primiparous N= 149,949**	**Model 2C.2 Multiparous N=113,569**
**5 joint chronic hypertension and hypertensive disorders developing during pregnancy:**	aRR (95% CI)	aRR (95% CI)	aRR (95% CI)
Chronic hypertension with superimposed preeclampsia	4.97 (4.46-5.54)	4.50 (3.93-5.15)	5.89 (4.91-7.05)
Chronic hypertension and no preeclampsia	1.17 (1.00-1.36)	0.98 (0.79-1.22)	1.41 (1.14-1.75)
No chronic hypertension with preeclampsia	5.12 (4.79-5.48)	5.02 (4.66-5.40)	5.38 (4.59-6.30)
Gestational hypertension[a]	1.78 (1.60-1.99)	1.77 (1.57-1.99)	1.78 (1.38-2.28)
No chronic hypertension and no preeclampsia or gestational hypertension (reference group)	1.0 (Referent)	1.0 (Referent)	1.0 (Referent)

[a] Gestational hypertension does not apply to the chronic hypertension group.

Covariates: Racial and ethnic groups, age, parity, pre-pregnancy BMI, diabetes status (pregestational, GDM, No diabetes), smoking habit, type of health insurance, and neighborhood deprivation index (NDI).

Entire Sample: No heart, kidney or liver disease, or malignancy cancer, before or during pregnancy.

© 2024 Gunderson EP et al. *JAMA Network Open.*

eTable9. Models 1-4: Unadjusted and Adjusted Relative Risks (95% Confidence Intervals) of Severe Maternal Morbidity (Without Blood Transfusion Indicator) for 2 Independent Variables for Hypertensive Disorders: Chronic Hypertension and Hypertensive Disorders Developing During Pregnancy.

**Entire Cohort n=263,518**		**Relative Risk of Severe Maternal Morbidity Without Blood Transfusion Indicator**			
**Hypertensive Conditions**	**SMM cases/ Total group**	**Model 1 Unadjusted**	**Models 2 Unadjusted**	**Model 3 Independently Adjusted**	**Model 4 Fully Adjusted plus Covariates**
**2 independent variables for hypertensive disorders of pregnancy**	**n/N (%)**	**Crude RR (95% CI)**	**Crude RR (95% CI)**	**aRR (95% CI)**	**aRR (95% CI)**
1) Chronic hypertension status:					
Chronic hypertension	364/13,626 (2.7)	2.31 (2.07-2.57)	--------------	1.07 (0.95-1.19)	1.03 (0.91-1.16)
No chronic hypertension	2,892/249,892 (1.2)	1.0 (Referent)	--------------	1.0 (Referent)	1.0 (Referent)
2) Hypertensive disorders developing during pregnancy:					
Any preeclampsia (with or without chronic hypertension)	988/16,071 (6.1)	--------------	7.00 (6.50-7.53)	6.89 (6.37-7.46)	6.27 (5.78-6.81)
Gestational hypertension[a]	195/11,514 (1.7)	--------------	1.93 (1.67-2.23)	1.93 (1.67-2.24)	1.82 (1.57-2.11)
No preeclampsia or gestational hypertension (with or without chronic hypertension)	2,073/235,933 (0.9)	--------------	1.0 (Referent)	1.0 (Referent)	1.0 (Referent)

[a] Gestational hypertension does not occur with chronic hypertension.

Covariates: Racial and ethnic groups, age, parity, pre-pregnancy BMI, diabetes status, smoking habit, type of health insurance, and NDI.

Entire Sample: No heart, liver, or kidney disease, or malignancy cancer, and no alcoholic liver disease during pregnancy or prior to pregnancy.

© 2024 Gunderson EP et al. *JAMA Network Open.*

eTable10. Model 4: Fully Adjusted Relative Risks (95% Confidence Intervals) of Severe Maternal Morbidity (Without Blood Transfusion Indicator) for 2 Independent Variables for Hypertensive Disorders: Chronic Hypertension and Hypertensive Disorders Developing During Pregnancy with Covariates.

**Entire Cohort (n=263,518)**	**Relative Risk of SMM Without Blood Transfusion Cases**	
**Model 4 = Fully Adjusted Model with covariates 2 independent variables for hypertensive disorders of pregnancy**	**aRR (95% CI)**	**n/N (%)**
1) Chronic hypertension status:		
Chronic hypertension	1.03 (0.91-1.16)	364/13,626 (2.7)
No chronic hypertension	1.0 (Referent)	2,892/249,892 (1.2)
2) Hypertensive disorders developing during pregnancy:		
Any preeclampsia (with or without chronic hypertension)	6.27 (5.78-6.81)	988/16,071 (6.1)
Gestational hypertension[a]	1.82 (1.57-2.11)	195/11,514 (1.7)
No preeclampsia or gestational hypertension (with or without chronic hypertension)	1.0 (Referent)	2,073/235,933 (0.9)
**Covariates**:		
Racial and Ethnic groups:		
Asian	1.37 (1.26-1.50)	960/66,090 (1.5)
American Indian/Native Alaskan	1.14 (0.60-2.18)	9/823 (1.1)
Black	1.42 (1.25-1.62)	319/19,985 (1.6)
Hispanic	1.20 (1.09-1.32)	824/69,297 (1.2)
Mixed/Not specified	1.01 (0.82-1.24)	95/8,883 (1.1)
Native Hawaiian/Pacific Islander	1.18 (0.80-1.74)	25/1,972 (1.3)
White	1.0 (Referent)	1,024/96,468 (1.1)
Age, year	1.04 (1.03-1.04)	-----
Parity		
Nulliparous (no prior births)	1.56 (1.44-1.69)	2,240/149,949 (1.5)
Parous (≥1 prior births)	1.0 (Referent)	1,016/113,569 (0.9)
Pre-pregnancy BMI, kg/m^2	1.0 (0.99-1.00)	----
Diabetes status		
Pregestational diabetes	1.29 (1.02-1.64)	74/2,470 (3.0)
Gestational diabetes	0.96 (0.86-1.06)	448/31,707 (1.4)
None	1.0 (Referent)	2,734/229,341 (1.2)
Neighborhood deprivation index		
≤ −1 (Least deprived)	1.0 (Referent)	362/29,921 (1.2)
> −1 and ≤ 0	1.06 (0.94-1.18)	1,651/132,667 (1.2)
>0 and ≤1	1.06 (0.93-1.20)	835/68,548 (1.2)
>1 (Most deprived)	1.10 (0.95-1.28)	404/31,953 (1.3)

© 2024 Gunderson EP et al. *JAMA Network Open.*

**eTable10.** Continued		
**Entire Cohort (n=263,518)**	**Relative Risk of SMM Without Blood Transfusion Cases**	
**Model 4 = Fully Adjusted Model with covariates 2 independent variables for hypertensive disorders of pregnancy**	**aRR (95% CI)**	**n/N (%)**
Type of health insurance		
Government: MediCal, Medicaid, State-subsidized	1.35 (1.19-1.53)	285/19,624 (1.5)
Employer/Self-funded/Other	1.0 (Referent)	2,971/243,894 (1.2)
Smoking (cigarette) habit		
Current	0.94 (0.80-1.09)	173/15,139 (1.1)
Former	0.97 (0.88-1.08)	409/32,536 (1.3)
Never	1.0 (Referent)	2,659/214,546 (1.2)
Unknown	0.90 (0.55-1.47)	15/1,297 (1.2)

[a] Gestational hypertension does not apply to the chronic hypertension group.

Entire Sample: No heart, liver, or kidney disease, or malignancy cancer, and no alcoholic liver disease during pregnancy or prior to pregnancy.

Covariates: Racial and ethnic groups, age, parity, pre-pregnancy BMI, diabetes status, smoking habit, type of health insurance, and NDI.

© 2024 Gunderson EP et al. *JAMA Network Open.*

eTable11. Models 1C and 2C: Unadjusted and Adjusted Relative Risks (95% Confidence Intervals) of Severe Maternal Morbidity (Without Blood Transfusion Indicator) for 5 Joint Chronic Hypertension and Hypertensive Disorders Developing During Pregnancy Subgroups.

**Entire cohort n=263,518**	**Relative Risk of SMM Without Blood Transfusion**		
**Hypertensive Condition Groups**	**SMM cases/ Total group**	**Model 1C Unadjusted**	**Model 2C Fully adjusted**
**5 joint chronic hypertension and hypertensive disorders developing during pregnancy subgroups**	**n/N (%)**	**Crude RR (95% CI)**	**aRR (95%CI)**
Chronic hypertension with superimposed preeclampsia	282/4,297 (6.6)	7.47 (6.62-8.43)	6.57 (5.75-7.50)
Chronic hypertension and no preeclampsia	82/9,329 (0.9)	1.03 (0.80-1.25)	0.95 (0.76-1.19)
No chronic hypertension with preeclampsia	706/11,774 (6.0)	6.82 (6.28-7.42)	6.19 (5.67-6.76)
Gestational hypertension[a]	195/11,514 (1.7)	1.93 (1.67-2.23)	1.81 (1.56-2.10)
No chronic hypertension and no preeclampsia or gestational hypertension (reference group)	1,991/226,604 (0.9)	1.0 (Referent)	1.0 (Referent)

[a] Gestational hypertension does not apply to the chronic hypertension group.

Covariates: Racial and ethnic groups, age, parity, pre-pregnancy BMI, diabetes status, smoking habit, type of health insurance, and NDI.

© 2024 Gunderson EP et al. *JAMA Network Open.*

eTable12. Model 2C: Fully Adjusted Relative Risks (95% Confidence Intervals) of Severe Maternal Morbidity (Without Blood Transfusion Indicator) for 5 Joint Chronic Hypertension and Hypertensive Disorders Developing During Pregnancy Subgroups with Covariates.

Entire cohort (n=263,518)	Relative Risk of Severe Maternal Morbidity Without Blood Transfusion	
**Model 2C Fully Adjusted with Covariates**	**aRR (95% CI)**	**n/N (%)**
5 joint chronic hypertension and hypertensive disorders developing during pregnancy subgroups:		
Chronic hypertension with superimposed preeclampsia	6.57 (5.75-7.50)	282/4,297 (6.6)
Chronic hypertension and no preeclampsia	0.95 (0.76-1.19)	82/9,329 (0.9)
No chronic hypertension with preeclampsia	6.19 (5.67-6.76)	706/11,774 (6.0)
Gestational hypertension[a]	1.81 (1.56-2.10)	195/11,514 (1.7)
No chronic hypertension and no preeclampsia or gestational hypertension (reference group)	1.0 (Referent)	1,991/226,604 (0.9)
Covariates:		
Racial and Ethnic groups		
Asian	1.37 (1.25-1.50)	960/66,090 (1.5)
American Indian/Native Alaskan	1.14 (0.60-2.18)	9/823 (1.1)
Black	1.42 (1.25-1.62)	319/19,985 (1.6)
Hispanic	1.20 (1.09-1.32)	824/69,297 (1.2)
Mixed/Not specified	1.01 (0.82-1.24)	95/8,883 (1.1)
Native Hawaiian/Pacific Islander	1.18 (0.80-1.74)	25/1,972 (1.3)
White	1.0 (Referent)	1,024/96,468 (1.1)
Age, y	1.04 (1.03-1.04)	
Parity		
Nulliparous (no prior births)	1.56 (1.44-1.69)	2,240/149,949 (1.5)
Parous (≥1 prior births)	1.0 (Referent)	1,016/113,569 (0.9)
Prepregnancy BMI, kg/m^2	1.00 (0.99-1.00)	-
Diabetes status		
Pregestational diabetes	1.29 (1.02-1.63)	74/2,470 (3.0)
Gestational diabetes	0.96 (0.86-1.06)	448/31,707 (1.4)
None	1.0 (Referent)	2,734/229,341 (1.2)
Neighborhood deprivation index		
≤−1 (Least deprived)	1.0 (Referent)	362/29,921 (1.2)
>−1 and ≤ 0	1.06 (0.94-1.18)	1,651/132,667 (1.2)
>0 and ≤1	1.06 (0.93-1.20)	835/68,548 (1.2)
>1 (Most deprived)	1.10 (0.95-1.28)	404/31,953 (1.3)
Type of health insurance		
Government: MediCal, Medicaid, State-subsidized	1.35 (1.19-1.53)	285/19,624 (1.5)
Employer/Self-funded/Other	1.0 (Referent)	2,971/243,894 (1.2)

© 2024 Gunderson EP et al. *JAMA Network Open.*

eTable12. Continued

**Entire cohort (n=263,518)**	**Relative Risk of Severe Maternal Morbidity Without Blood Transfusion**	
**Model 2C Fully Adjusted with Covariates**	**aRR (95% CI)**	**n/N (%)**
5 joint chronic hypertension and hypertensive disorders developing during pregnancy subgroups:		
Smoking (cigarette) habit		
Current	0.94 (0.80-1.09)	173/15,139 (1.1)
Former	0.97 (0.88-1.08)	409/32,536 (1.3)
Unknown	0.90 (0.55-1.47)	15/1,297 (1.2)
Never	1.0 (Referent)	2,659/214,546 (1.2)

[a] Gestational hypertension does not apply to the chronic hypertension group.

Covariates: Racial and ethnic groups, age, parity, pre-pregnancy BMI, diabetes status, smoking habit, type of health insurance, and NDI.

© 2024 Gunderson EP et al. *JAMA Network Open.*

eFigure 1. Selection Criteria to Identify One Singleton Live or Stillbirth Per Woman from 2009 to 2019.

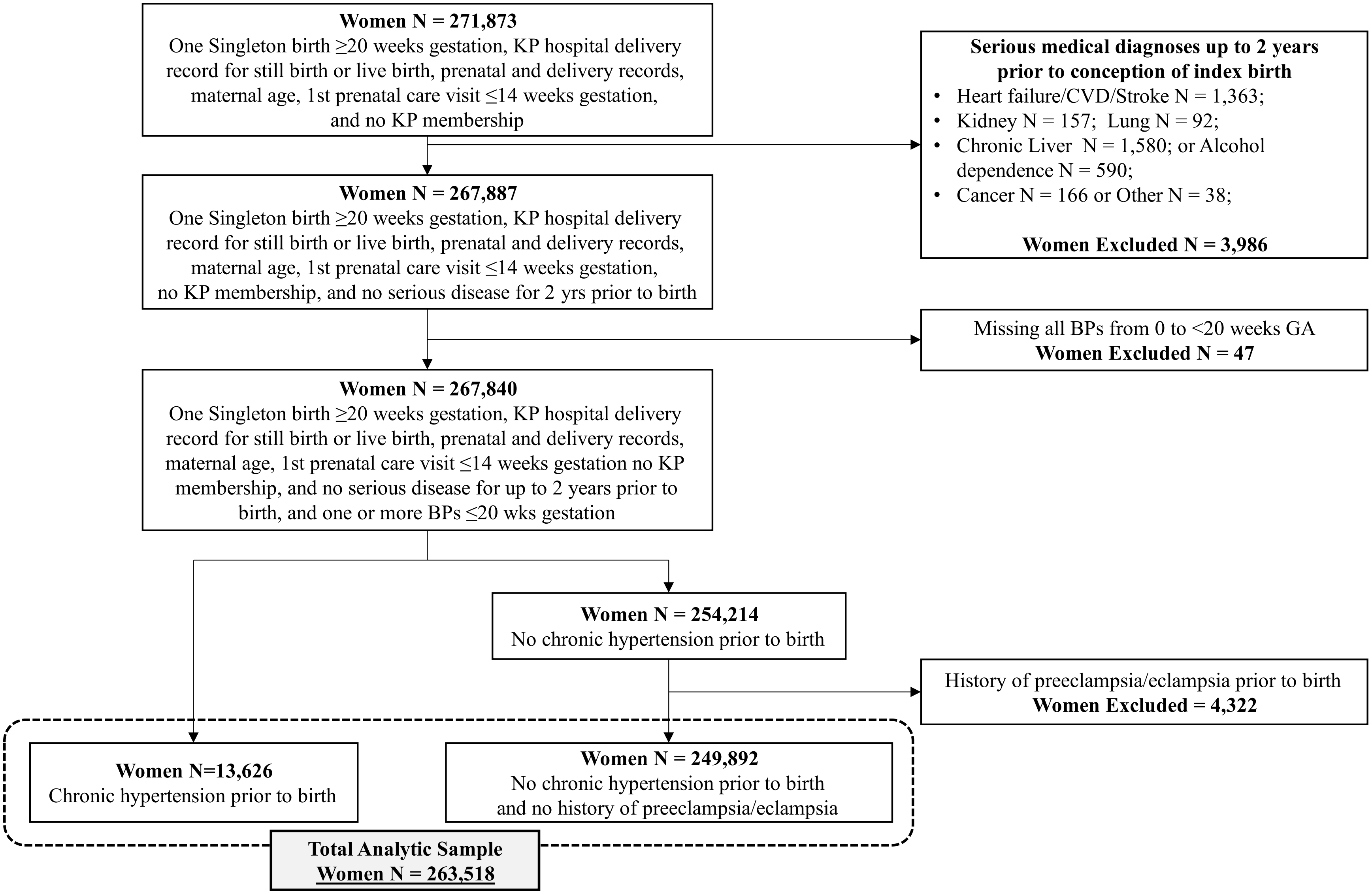

© 2024 Gunderson EP et al. *JAMA Network Open.*

eFigure2. Directed Acyclic Graph (DAG) – Causal and Non-causal paths

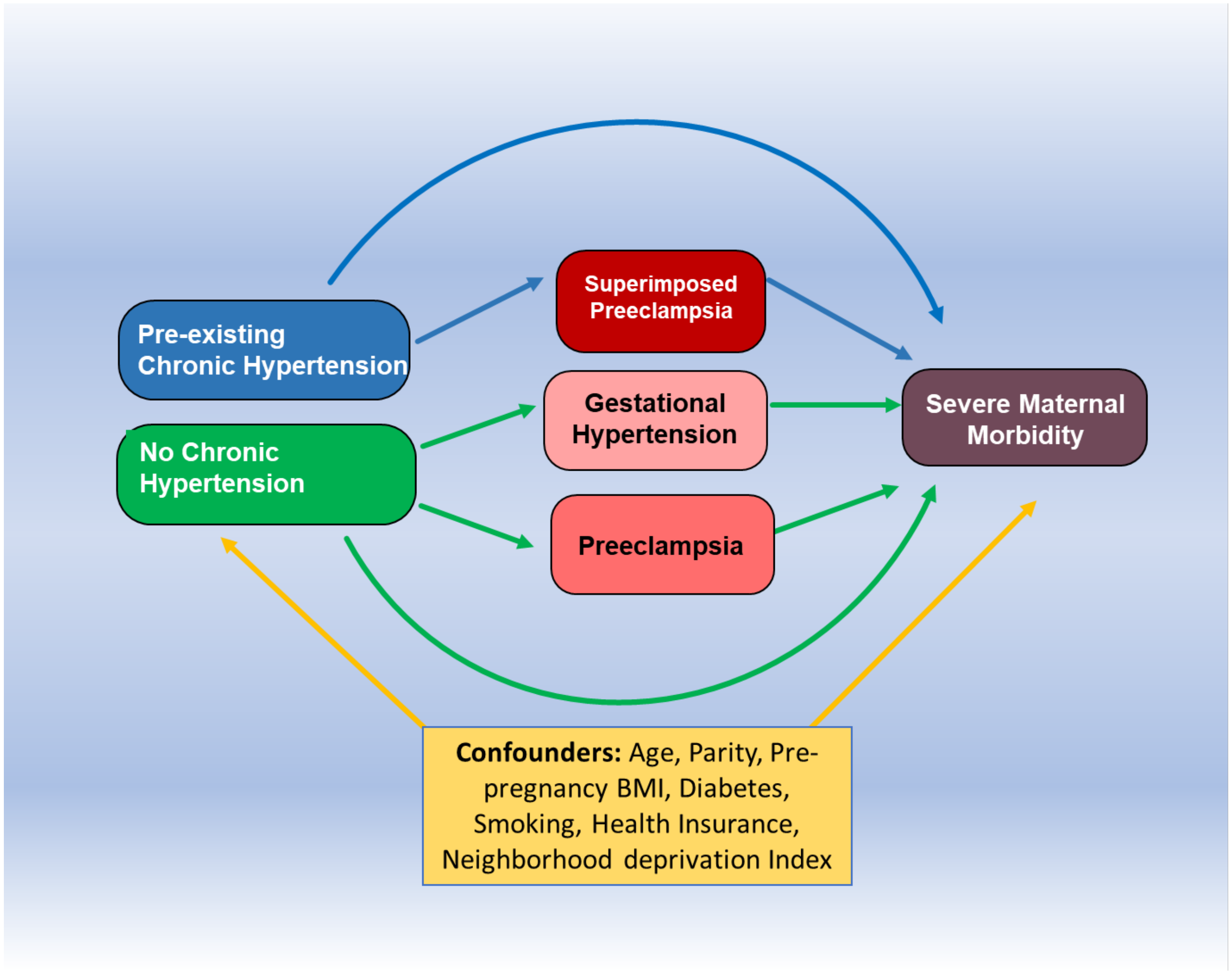

Causal paths linking Chronic Hypertension and Severe Maternal Morbidity
Pre-pregnancy Chronic Hypertension ➡ Severe Maternal Morbidity
Pre-pregnancy Chronic Hypertension ➡ Superimposed Preeclampsia ➡ Severe Maternal Morbidity

Non-causal paths linking Chronic Hypertension and Severe Maternal Morbidity
Pre-pregnancy Chronic Hypertension ⬅ Confounders ➡ Gestational Hypertension ➡ Severe Maternal Morbidity
(block by controlling for covariates, including Racial and ethnic groups, age, parity, pre-pregnancy BMI, diabetes status, smoking habit, type of health insurance, and neighborhood deprivation index (NDI)).

© 2024 Gunderson EP et al. *JAMA Network Open.*

eFigure 3. Risk Differences for Severe Maternal Morbidity Rates Minus Rates for the Respective Referent Groups.

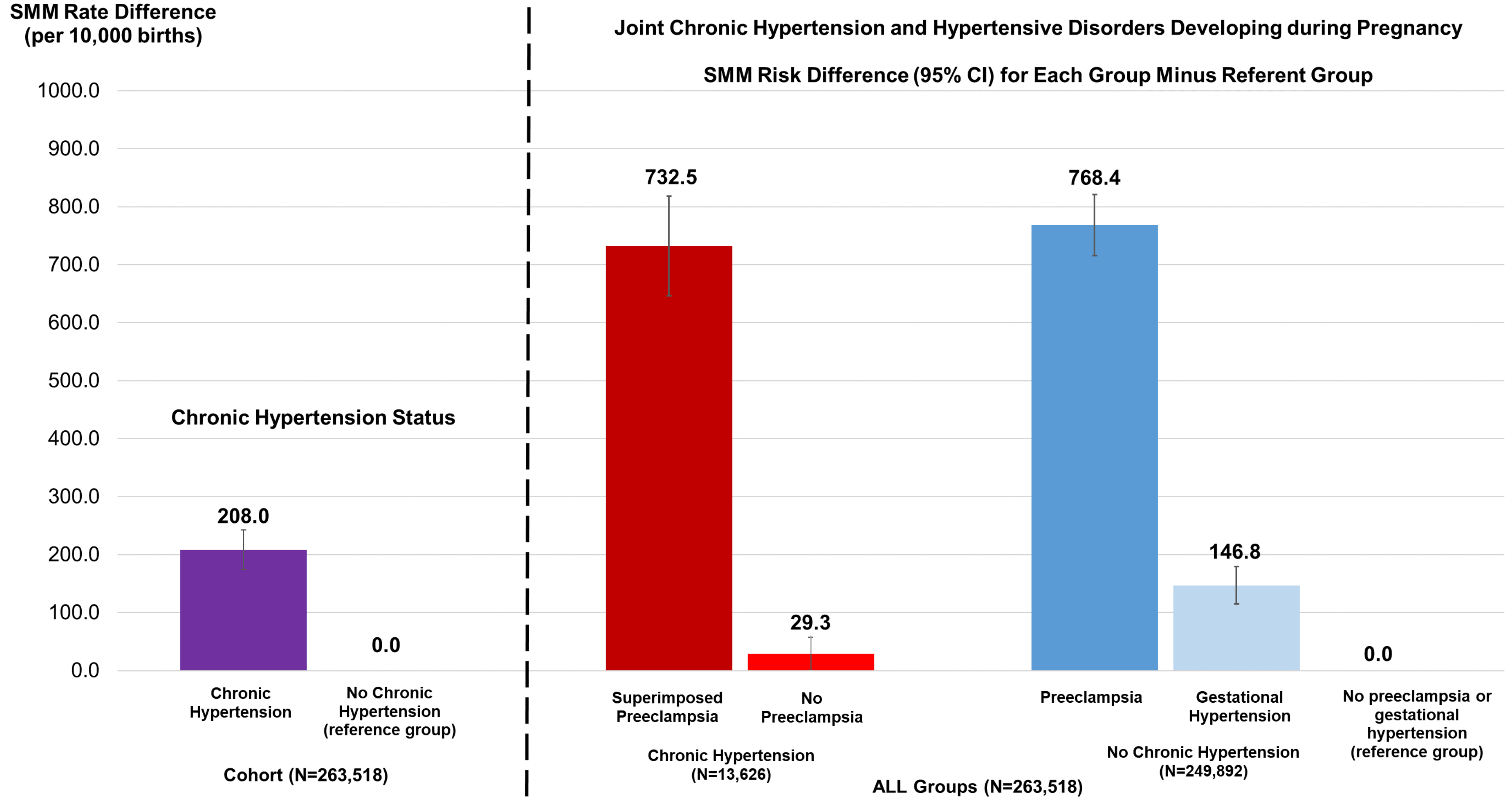

© 2024 Gunderson EP et al. *JAMA Network Open.*
