# Supplementary material for: Severe Maternal Morbidity Associated With Chronic Hypertension, Preeclampsia, and Gestational Hypertension
Source: JAMA Netw Open. 2025 Jan 28;8(1):e2451406. doi: 10.1001/jamanetworkopen.2024.51406 (PMC11775729; doi:10.1001/jamanetworkopen.2024.51406)
Supplement: Supplement 2. — Data Sharing Statement [file jamanetwopen-e2451406-s002.pdf]

Data Sharing Statement

Gunderson. Severe Maternal Morbidity Associated With Chronic Hypertension, Preeclampsia, and Gestational Hypertension. *JAMA Netw Open*. Published , 2024.
doi:10.1001/jamanetworkopen.2024.51406

Data
Data available: No

Additional Information
Explanation for why data not available: Requests to access the dataset from qualified researchers trained in human subjects' confidentiality protocols may be sent to Dr. Erica P. Gunderson, Principal Investigator, at the Division of Research, email: erica.gunderson@kp.org. The patient data is owned by the Kaiser Foundation Health Plan, Inc., Kaiser Foundation Hospitals, Inc., and The Permanente Medical Group, Inc. Because of their third-party rights, it is not possible to make the data publicly available without restriction.
